# Differential Proteome Profiling Using iTRAQ in Microalbuminuric and Normoalbuminuric Type 2 Diabetic Patients

**DOI:** 10.1155/2012/168602

**Published:** 2012-03-27

**Authors:** Jonghwa Jin, Yun Hyi Ku, Yikwon Kim, Yeonjung Kim, Kyunggon Kim, Ji Yoon Lee, Young Min Cho, Hong Kyu Lee, Kyong Soo Park, Youngsoo Kim

**Affiliations:** ^1^Department of Biomedical Sciences, Seoul National University College of Medicine, 28 Yongon-Dong, Seoul 110-799, Republic of Korea; ^2^Department of Internal Medicine, Seoul National University College of Medicine, 28 Yongon-Dong, Seoul 110-799, Republic of Korea; ^3^National Instrumentation Center for Environmental Management, Seoul National University, Seoul 151-921, Republic of Korea; ^4^Genome Research Center for Diabetes and Endocrine Disease, Seoul National University College of Medicine, 28 Yongon-Dong, Seoul 110-799, Republic of Korea

## Abstract

Diabetic nephropathy (DN) is a long-term complication of diabetes mellitus that leads to end-stage renal disease. Microalbuminuria is used for the early detection of diabetic renal damage, but such levels do not reflect the state of incipient DN precisely in type 2 diabetic patients because microalbuminuria develops in other diseases, necessitating more accurate biomarkers that detect incipient DN. Isobaric tags for relative and absolute quantification (iTRAQ) were used to identify urinary proteins that were differentially excreted in normoalbuminuric and microalbuminuric patients with type 2 diabetes where 710 and 196 proteins were identified and quantified, respectively. Some candidates were confirmed by 2-DE analysis, or validated by Western blot and multiple reaction monitoring (MRM). Specifically, some differentially expressed proteins were verified by MRM in urine from normoalbuminuric and microalbuminuric patients with type 2 diabetes, wherein alpha-1-antitrypsin, alpha-1-acid glycoprotein 1, and prostate stem cell antigen had excellent AUC values (0.849, 0.873, and 0.825, resp.). Moreover, we performed a multiplex assay using these biomarker candidates, resulting in a merged AUC value of 0.921. Although the differentially expressed proteins in this iTRAQ study require further validation in larger and categorized sample groups, they constitute baseline data on preliminary biomarker candidates that can be used to discover novel biomarkers for incipient DN.

## 1. Introduction

 Diabetes mellitus is a chronic disease with potentially devastating complications. For example, diabetes mellitus is associated with macrovascular complications, such as cardiovascular and cerebrovascular diseases, and microvascular complications, including diabetic nephropathy (DN) and retinopathy [1]. DN is a long-term complication of diabetes that is caused by specific renal structural alterations, such as mesangium expansion due to the progressive accumulation of extracellular matrix (ECM), and by functional losses, such as elevated glomerular basement membrane (GBM) permeability [2].

DN occurs in 15% to 25% of type 1 diabetic patients and 30% to 40% of type 2 diabetic patients [3] and accounts for approximately one-half of all new cases of end-stage renal disease (ESRD). Furthermore, ESRD has a 5-year survival rate of only 21% [4]. Because the progression of ESRD in DN is irreversible, the early diagnosis of DN is necessary to prevent or delay progression to ESRD [5]. Microalbuminuria represents a potentially reversible incipient stage of nephropathy and is used as a noninvasive index for the detection of diabetic renal disease. Microalbuminuria is defined as a state in which abnormal amounts of albumin are excreted in urine (30–300 mg/24 h versus <30 mg/24 h in normoalbuminuria) [5, 6].

 The use of microalbuminuria to predict incipient DN, particularly in type 2 diabetic patients, is limited for several reasons [7]: the microalbuminuric state also predicts cardiovascular disease in diabetic and nondiabetic individuals [8, 9], and it is associated with inflammation and hypertension [5]. Consequently, the likelihood of detecting nondiabetic renal disease or normal glomerular structure is observed with microalbuminuria patients [10]. Thus, more accurate biomarkers for incipient DN in type 2 diabetic patients are required that can differentiate incipient DN from other conditions in microalbuminuria patients, including cardiovascular disease, inflammation, and hypertension.

 Recently, to compare DN patients with non-DN patients, proteomic technologies have been developed to identify urinary marker candidates that are associated with the development of DN. Various proteomic approaches have been used for this purpose, including 2-DE, 2-DE DIGE, and SELDI-TOF [5, 11, 12]. However, because many studies have focused on restricted sets of targeted proteins, alterations in comprehensive urinary protein profiles in type 2 diabetes have not been monitored. In particular, SELDI-TOF has been shown to be a valuable technology for urinary proteomic analysis, but the absolute identification of differentially excreted proteins remains challenging [13].

 To scan a comprehensive differential proteome for preliminary DN candidate biomarkers, we used a 4-plex isobaric tag for relative and absolute quantification (iTRAQ, 4-plex), allowing us to identify and quantify proteins in up to 4 samples [14]. The advantages of iTRAQ include whole labeling of representative or pooled samples, comparatively high throughput, and retention of posttranslational modification (PTM) data; one of its shortcomings is that it cannot be applied easily to a large collection of individual clinical samples due to reagent cost and the required mass spectrometry effort [15]. To date, iTRAQ has been applied to a variety of sample sets, including *E. coli*, mammalian cells, yeast, plant cells, and human biological fluids [16–22].

Therefore, in this study, we used iTRAQ to identify and quantify differentially excreted urinary proteins in microalbuminuric versus normoalbuminuric type 2 diabetic patients and investigate the associations that would reflect the progress of DN. Afterward, those differentially excreted urinary proteins have been confirmed by 2-DE, followed by MALDI-TOF/TOF, or validated by Western blot and MRM.

## 2. Materials and Methods

### 2.1. Urine Sample Preparation

Type 2 diabetic subjects (age ≥ 40 years) with or without microalbuminuria who were patients at the Diabetes Center of Seoul National University Hospital, Seoul, Republic of Korea, were enrolled in 2006. Microalbuminuria patients were randomly selected out of these outpatients, whereas normoalbuminuric patients were selected to be matched to age, sex, body mass index (BMI), and DM duration with microalbuminuric patients.

Forty-three subjects with diabetic retinopathy and persistent microalbuminuria formed the microalbuminuria group (MA). Persistent microalbuminuria was defined as an albumin : creatinine ratio (ACR) between 30 and 300 mg/g in 2 urine samples that were taken over 3 months. The normoalbuminuria group (NA) comprised subjects who had no diabetic retinopathy, did not use angiotensin inhibitors or angiotensin receptor blockers that lowered albuminuria, and showed no microalbuminuria in their urine in the past year (urinary albumin < 30 mg/g creatinine). Forty-three subjects formed the NA group.

There were no significant differences in age, sex, body mass index, or diabetes mellitus duration between the 2 study groups. Subjects with hematuria, uncontrolled hypertension (blood pressure ≥ 140/90 mm Hg), uncontrolled hyperglycemia (glycated hemoglobin A1c ≥ 8.5%), urinary tract infection, acute febrile illness, congestive heart failure, or malignancy were excluded. Individuals who were receiving peroxisome proliferator-activated receptor gamma agonists were also excluded. Midstream urine of spot urine samples were collected in sterile 50-mL tubes that contained 50 *μ*L 0.1 mM PMSF (serine protease inhibitor) and 500 *μ*L 1 mM sodium azide from 86 patients and were stored at −80°C until use. Informed consent was obtained from all subjects after obtaining approval for the study from the Institutional Review Board at Seoul National University Hospital.

Urine albumin and creatinine were measured in spot urine samples by immunoturbidimetric method using the TIA Micro Alb Kit (Nittobo, Tokyo, Japan) and enzymatic creatinine assay (Roche, Mannheim, Germany), respectively, on a Hitachi 7170 autoanalyzer (Hitachi, Tokyo, Japan).

For the iTRAQ and 2-DE experiments, pooled urine samples, based on average albumin-to-creatinine ratios, were used; the clinical characteristics of the study subjects are summarized in Table 1. Because the protein concentration of each urine sample varied widely, depending on the urine volume in the morning, equal amounts of total protein from each patient were pooled to prepare the urine samples (NA1–NA4 and MA1–MA4).

To prepare the protein samples, approximately 50 mL aliquots of normoalbuminuric and microalbuminuric urine were centrifuged at 3000 g for 30 min at 4°C. Supernatants were filtered through a 0.22 *μ*m MILLEX GP membrane (Millipore, Carrigtwohill, Cork, Ireland) and concentrated to 3 mL in an Amicon ultrafiltration cell (YM2, 3 kDa MW cut-off, Millipore). The concentrated urine samples were then desalted by dialysis twice using a Slide-A-Lyzer dialysis cassette kit (3.5 kDa, Pierce, Rockford, ILUSA) against 1000 volumes of distilled water, containing 0.1 mM PMSF (serine protease inhibitor) and 1 mM *β*-ME, at 4°C. Proteins in the dialyzed urine were precipitated with 5 volumes of acetone for 4 hrs at −20°C, and the resulting pellets were washed 3 times with cold acetone; the supernatants were discarded.

### 2.2. Labeling with iTRAQ Reagents

 Aliquots of 100 *μ*g of protein were reduced, alkylated, digested, and labeled according to the manufacturer's instructions (Applied Biosystems, Foster City, CA, USA). Briefly, 1 *μ*L of denaturant (2% SDS) and 1 *μ*L of reducing reagent (50 mM tris-[2-carboxyethyl] phosphine) were added to each sample and incubated for 1 hr at 60°C. Each sample was allowed to cool at room temperature, and 1 *μ*L of cysteine blocking reagent (200 mM methyl methanethiosulfonate (MMTS) in isopropanol) was added and incubated for 20 min at room temperature. The tubes were digested with trypsin (Promega, Madison, WI, USA) at a protein-to-enzyme ratio of 10 : 1 at 37°C overnight, and the contents of one vial of iTRAQ reagent, dissolved in 70 *μ*L of ethanol, were added to each peptide mixture and incubated for 1 hr at room temperature.

**Figure 1:**
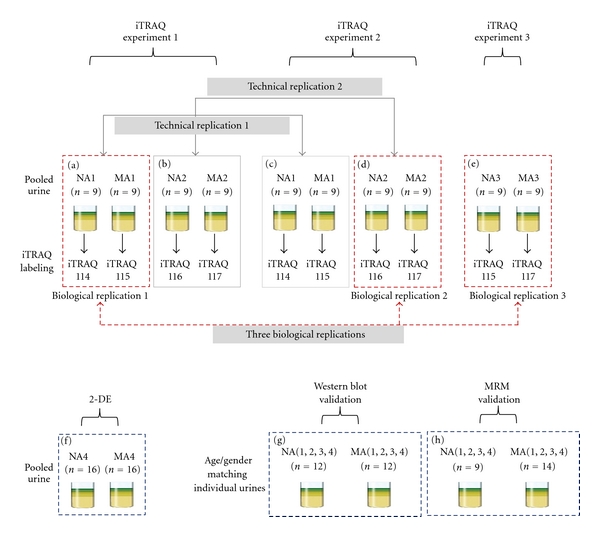
Workflow of iTRAQ, 2-DE, Western blot, and MRM of the urinary proteome. For analysis of the urinary proteome, 3 iTRAQ experiments were performed, 2-DE, Western blot, and MRM were conducted to confirm and validate the iTRAQ results. iTRAQ experiments 1, 2, and 3 were performed, labeled (a) and (b), (c) and (d), and (e), respectively, wherein 3 biological replicates (labeled (a), (d), and (e), resp.), technical replicate 1 (labeled (a) and (c)), and technical replicate 2 (labeled (b) and (d)) were performed in microalbuminuric versus normoalbuminuric urine. 2-DE, Western blot, and MRM analysis of the urinary proteome were conducted using labeled (f), (g), and (h), respectively.

In this study, 3 iTRAQ experiments were performed. The detailed iTRAQ labeling strategy is summarized for the specified NA/MA urine samples in Figure 1 and Table 1; iTRAQ Experiments 1, 2, and 3 were performed for labeling (a) and (b), (c) and (d), and (e), respectively. Each normoalbuminuric peptide was labeled with iTRAQ reagents 114, 115, and 116, and the microalbuminuric peptide was labeled with iTRAQ reagents 115 and 117 (Figure 1). The 2 sample sets (microalbuminuric and normoalbuminuric) were combined and dried. To analyze the proteome quantitatively using iTRAQ labeling, we determined the labeling efficiency, as described [23]; the number of possible labeling sites (the N-termini of all peptides and lysine side chains) in 21,610 peptides were compared manually with that of completely labeled sites, represented by the Pro GroupTM Algorithm in ProteinPilot.

### 2.3. Strong Cation Exchange Chromatographic Fractionation

iTRAQ-labeled samples were subjected to LC-MS/MS at the National Instrumentation Center for Environmental Management, Seoul National University, and fractionated using strong cation exchange (SCX) chromatography, as follows. Dried samples were reconstituted in 500 *μ*L of buffer A (25% v/v acetonitrile (ACN) and 5 mM ammonium formate, adjusted to pH 2.7 with formic acid) and loaded onto a PolySULFOETHYL A column (4.6 mm id × 100 mm, 5 *μ*m, 200 Å; PolyLC, Columbia, MD, USA) in a HP1100 series HPLC (Agilent Technologies, Palo Alto, CA, USA). The column was equilibrated for 5 min in buffer A, and the peptides were eluted using a gradient of 0% to 30% buffer B (25% v/v ACN and 1 M ammonium formate [pH 3] with formic acid) over 80 min and 30% to 90% buffer B for 40 min at a flow rate of 0.7 mL/min. Absorbance was monitored at 280 nm, and the fractions were collected every 2 min after injection.

### 2.4. LC-MS/MS Analysis

 Fractions were reconstituted in solvent A and injected into an LC-ESI-MS/MS system. LC-MS/MS was performed using an integrated system, which consisted of an autosampler switching pump and a micropump (Tempo Nano LC system; Applied Biosystems) with a hybrid quadrupole-TOF LC-MS/MS spectrometer (QStar Elite; Applied Biosystems) that was equipped with a nanoelectrospray ionization source (Applied Biosystems) and fitted with a 10 *μ*m fused silica emitter tip (New Objective, Woburn, MA, USA).

Peptides were first trapped on a Zorbax 300SB-C18 trap column (300 *μ*m id × 5 mm, 5 *μ*m, 100 Å; Agilent Technologies), washed for 10 min with 98% solvent A (water/ACN [98 : 2 v/v] and 0.1% formic acid) and 2% solvent B (water/ACN [2 : 98 v/v] and 0.1% formic acid) at a flow rate of 10 *μ*L/min, and separated on a Zorbax 300SB-C18 capillary column (75 *μ*m id × 150 mm, 3.5 *μ*m, 100 Å) at a flow rate of 300 nL/min. The LC gradient was run at 2% to 35% solvent B over 120 min and from 35% to 90% over 10 min, followed by 90% solvent B for 15 min, and finally 5% solvent B for 35 min. The resulting peptides were electrosprayed through a coated silica tip (New Objective) at an ion spray voltage of 2300 eV.

For data acquisition, the mass spectrometer was set in the positive ion mode at a selected mass range of 400–1600 *m/z *for a 1 sec TOF-MS survey scan to detect precursor ions. The 5 most abundant peptides (count >20) with charge states of +2 to +4 were selected to perform the information-dependent acquisition (IDA) of MS/MS data. Once selected, the precursor ions were dynamically excluded for 60 sec at a mass tolerance of 100 ppm.

### 2.5. Data Analysis

 Data file processing, protein identification, and relative abundance quantification were performed using ProteinPilot v.2.0.1 (Applied Biosystems; MDS-Sciex, Concord, Canada) and the Paragon algorithm [24]. Database searches were performed against the Celera human database (human KBMS 5.0, 2005-03-02; a total of 187,748 entries provided by Applied Biosystems). The search parameters used were: a peptide and fragment ion mass tolerance of 0.2 Da; 1 missed trypsin cleavage; fixed cysteine modification by MMTS; variable oxidation of methionine; and iTRAQ labeling of the N-termini of peptides and lysine side chain residues.

The confidence threshold for protein identification was an unused ProtScore >1.3 (95% confidence interval). ProteinPilot computes a percentage confidence that reflects the probability that a hit is a false positive; thus, at the 95% confidence level, the false positive identification rate is approximately 5% [24, 25]. Although this program automatically accepts all peptides that have a confidence level >1%, only proteins with at least 1 peptide that had a confidence level >95% were initially recorded. At these low confidence levels, peptides do not identify a single protein by themselves but, rather, support protein identification in the presence of other peptides [24, 25]. Quantification results were reported only when the error factor (EF) was <2, which indicates a standard deviation of quantification <20%.

### 2.6. GO Ontology Analysis

The “biological process” and “molecular function” classifications were analyzed using PANTHER ID numbers (http://www.pantherdb.org/), provided in the ProteinPilot output when a Celera human database is used. To construct a graphical representation of differentially excreted proteins, MultiExperiment Viewer (Version 4.3) was used, allowing us to generate a “heatmap” of differentially excreted proteomes (http://www.tm4.org/mev/).

### 2.7. 2-DE Urinary Proteome and PMF Analysis

Urine samples were pooled from 16 type 2 diabetic patients with normoalbuminuria and 16 type 2 diabetic patients with microalbuminuria. The characteristics of the pooled urine samples for 2-DE are shown in Figure 1 and Table 1. For the PMF analysis, a MALDI-TOF/TOF mass spectrometer (ABI 4700 Proteomics Analyzer, Applied Biosystems) was used as described in our previous papers [26, 27].

### 2.8. Western Blot Analysis

Twenty-four urine samples that were matched for gender and age (NA: 6 females and 6 males, and MA: 6 females; 6 males) were selected from the urine sample groups (NA1–NA4 and MA1–MA4, resp.) and subjected to Western blot validation of the 6 representative candidates from the iTRAQ experiments (Figures 1 and 6, and Table 1). The primary antibodies were directed against transferrin (1 : 500, AbFrontier, Seoul, Korea), ceruloplasmin (1 : 1000, AbFrontier), *α*1-antitrypsin (1 : 1000, AbFrontier), vitamin D-binding protein (1 : 1000, AbFrontier), *α*1-acid glycoprotein (1 : 2000, AbFrontier), and haptoglobin (1 : 1000, AbFrontier).

### 2.9. Candidate Validation Using Multiple Reaction Monitoring

In addition to Western blot, multiple reaction monitoring (MRM) was performed to verify the candidate biomarkers using 9 NA and 14 MA urine samples from the urine sample groups (NA1–NA4 and MA1–MA4, resp.) (Figure 1 and Table 1). In our MRM experiment [28], triple quadrupole linear ion trap MS (4000 Qtrap, coupled with a nano Tempo MDLC, Applied Biosystems) was performed; the detailed procedure is previously described [28]. Data were processed using the MultiQuant program (Applied Biosystems, version 1.0), and each peak area of the transitions was normalized to an input internal standard (Q1/Q3 transitions at 542.3/636.3 *m/z* for beta-galactosidase peptide) [28]. In the statistical analysis, receiver operating characteristic (ROC) curves and interactive plots were generated using Medicalc (MedCalc Software, Mariakerke, Belgium, version 10.0.1.0).

## 3. Results

### 3.1. Identification of Urinary Proteomes from Normoalbuminuric and Microalbuminuric Patients

For the iTRAQ experiments, 3 biological replicates (biological replicate 1 was labeled (a), replicate 2 was labeled (d), and replicate 3 was labeled (e)); 2 technical replicates (technical replicate 1 was labeled (a) and (c), replicate 2 was labeled (b) and (d)) were generated from normoalbuminuric and microalbuminuric urine (Figure 1). The 3 biological replicates were used to profile and quantitate the urinary proteome; the 2 technical replicates were used solely to determine the cutoff for significant fold-changes.

Seven hundred ten proteins were identified from 21,610 peptides of the 3 combined biological replicates at a minimum confidence level of 95% (unused ProtScore > 1.3). Of the proteins that were identified by iTRAQ, 27% comprised 1-peptide proteins; 14% was 2-peptide proteins; 8% was 3-peptide proteins; 5% was 4-peptide proteins; 46% comprised proteins that had 5 or more peptides. In our iTRAQ experiment, 83 proteins (unused ProtScore > 1.3) were common to all 3 biological replicates at a minimum confidence level of 95%, using 3 different pooled urine samples.

### 3.2. Determination of Cutoff for Significant Fold-Change in iTRAQ Experiments

 To generate the quantitative proteome using iTRAQ labeling, we first determined the labeling efficiency, which exceeded 98% (data not shown). Next, the cutoff for significant fold-change was determined based on the 2 technical replicates ((a) and (c) of iTRAQ experiment 1, (b); (d) of iTRAQ experiment 2) (Figure 1). In the 2 replicate experiments, the number of commonly identified proteins was 173 (2 [115/114] ratios from technical replicate 1) and 107 (2 [117/116] ratios from technical replicate 2), and the number of selected proteins was 44 (2 [115/114] ratios from technical replicate 1) and 26 (2 [117/116] ratios from technical replicate 2), which were chosen based on the following criteria: it contained more than 2 unique peptides (>95%), and *P* value <0.05 for the 115/114 and 117/116 reporter ions. The 70 proteins were used to monitor technical variations and confirm the threshold for meaningful differences.

**Figure 2:**
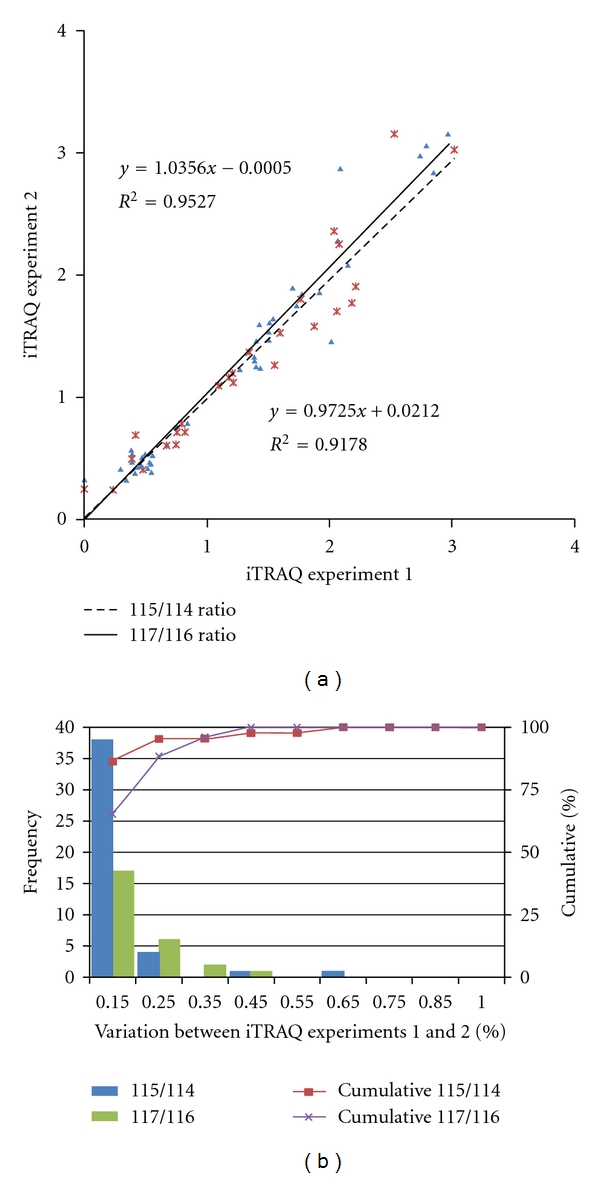
Correlation between the 2 technical replicates and determination of the cutoff value for significant fold changes. (a) Plots of iTRAQ ratios for two technical replicates. Forty-four proteins were commonly observed from technical replicate 1 (labeled 115/114), and 26 proteins were commonly observed from technical replicate 2 (labeled 117/116). These differentially excreted proteins (*P* value < 0.05, more than two unique peptides: >95%) were plotted in the linear dynamic range. The technical variations yielded a correlation coefficient of *r* ^2^ = 0.9527 and *r* ^2^ = 0.9178 between iTRAQ experiments 1 and 2, respectively. (b) The % variations for the common proteins from the two technical replicates. The 44 and 26 common proteins from the 2 technical replicates were used as inputs to calculate % variations. The vertical axis represents the number of proteins, and the horizontal axis denotes % variation. Ninety percent of the proteins fell within 25% of the respective experimental variation. Thus, we considered a fold-change of >1.25 or <0.80, a meaningful cutoff that represented actual differences in the iTRAQ experiments.

 The technical variations for the 115/114 and 117/116 reporter ions, calculated using the ratios of the 44 and 26 commonly observed proteins between the 2 technical replicates, were *r^2^* = 0.9527 and *r^2^* = 0.9178, respectively (Figure 2(a)). Accordingly, 90% of the commonly observed in the technical replicates fell within 25% of the respective experimental variation (Figure 2(b)). Therefore, we set fold-change thresholds of >1.25 or <0.80 to identify true differences between the expression of 115/114 and 116/117 reporter ions, as described in [23].

### 3.3. Differential Proteomes between Microalbuminuria and Normoalbuminuria

In our iTRAQ study, we obtained diverse biomarker candidates from 3 pooled biological NA/MA urine samples (each pooled NA or MA urine sample consisted of 9 individual urine specimens; thus, the 3 pooled NA, and 3 pooled MA urine samples comprised 54 different individual urine samples). Further, biomarker candidates were confirmed and validated by 2-DE, Western blot, and MRM.

To analyze urinary proteomes in normoalbuminuria and microalbuminuria subjects, 3 biological replicates were generated, wherein 196 proteins met the following criteria: *P* value < 0.05, EF < 2, more than 2 unique peptides with >95% confidence level, and protein expression >1.25 or <0.80 for all reporter ions; 99 and 97 proteins were upregulated and downregulated, respectively (Appendix A).

**Figure 3:**
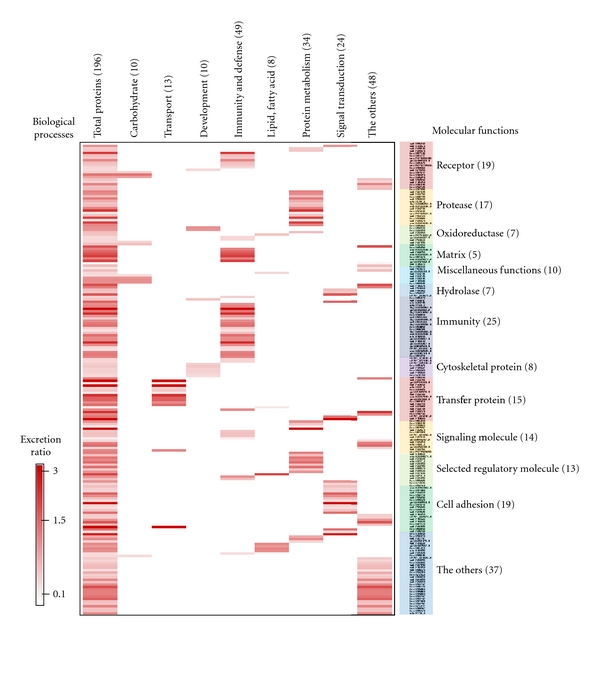
Comprehensive functional annotation of the differentially excreted proteome. The 196 quantitated urinary proteins were annotated for the “biological process” (*x*-axis) and “molecular function” subcategories (*y*-axis) in a heatmap. The “biological process” and “molecular function” categories comprised 8 and 13 subcategories, respectively. The 196 quantitated urinary proteins are individually assigned into the “biological process” and “molecular function” subcategories.

These proteins were further analyzed by differential proteomic expression. All quantified proteins were classified into “biological process” and “molecular function” subcategories using the PANTHER classification program, allowing us to analyze phenotypic features and molecular functions between microalbuminuria and normoalbuminuria (Figure 3). Moreover, to visualize the comprehensive functional annotations graphically, such as in a heatmap, the 196 proteins were first categorized by “biological process,” and a second dimension was added to coordinate the “molecular function” subcategories (Figure 3). The 196 proteins constituted a preliminary list of biomarkers; Figure 3 shows the expression ratios of the iTRAQ dataset and differentially excreted proteins in microalbuminuria versus normoalbuminuria urine.

### 3.4. Classification of Urinary Proteomes in Microalbuminuria versus Normoalbuminuria

**Figure 4:**
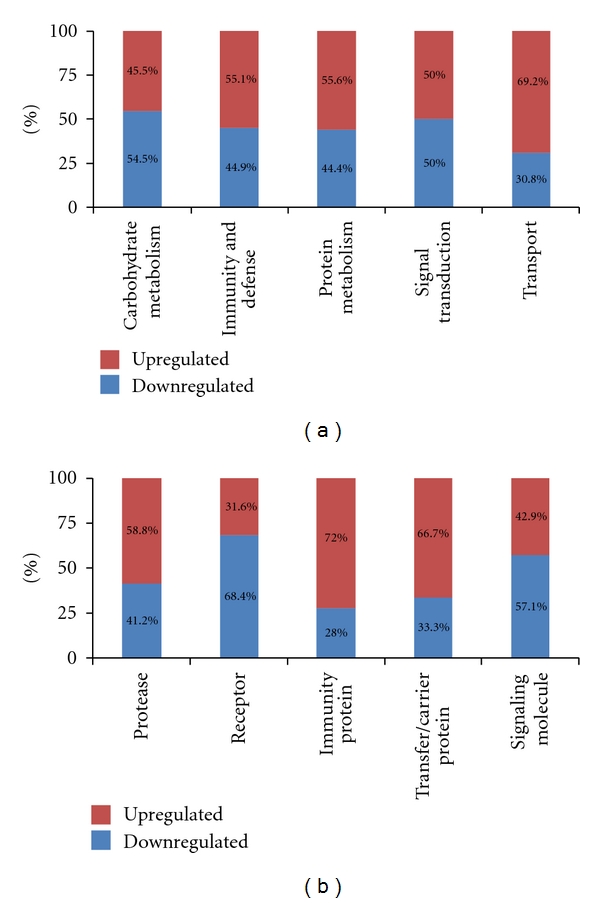
Functional distribution of differentially excreted proteins in microalbuminuria versus normoalbuminuria. Functional classification of differentially excreted proteins into (a) “biological process” and (b) “molecular function.” Only 5 major subcategories for “biological process” and “molecular function” are shown; each subcategory is presented as the percentage of up- and down-regulated proteins.

The 196 proteins from the 3 biological replicates were categorized by PANTHER ID number into “biological process” and “molecular function” groups; certain subcategories are summarized in Figures 4(a) and 4(b).

The “biological process” subcategories accounted for 196 differentially excreted proteins, wherein “immunity and defense” and “protein metabolism” represented 49 and 34 of the quantitated proteins, respectively—the 2 largest components (Figure 3). Moreover, in the “biological process” subcategories, “carbohydrate metabolism” (54.5%) and “signal transduction” (50%) were downregulated in microalbuminuric versus normoalbuminuric urine (Figure 4(a)). In contrast, 69.2%, 55.6%, and 55.1% of the 196 proteins were upregulated in the “transport,” “protein metabolism,” and “immunity and defense” subcategories, respectively (Figure 4(a)).

The “molecular function” subcategories accounted for 196 differentially excreted proteins, in which “immunity” and “receptor” represented 25 and 19 of the quantitated proteins, respectively—the 2 largest components (Figure 3). In the “molecular function” subcategories, “receptor” (68.4%) and “signaling molecule” (57.1%) proteins were down-regulated in microalbuminuric versus normoalbuminuric urine (Figure 4(b)). In contrast, 72.0%, 58.8%, and 66.7% of the 196 proteins were up-regulated in the “immunity protein,” “protease,” and “transfer/carrier protein” subcategories, respectively (Figure 4(b)).

### 3.5. Differentially Excreted Urinary Proteins Are Associated with Pathogenic Status

 One hundred ninety-six tentative biomarker candidates were differentially expressed, based on the iTRAQ data, and they were characterized biologically according to “biological process” and “molecular function.” Moreover, in a detailed association study of diabetic nephropathy and differentially excreted proteins using references and databases, we prioritized 10 candidates, of the 196 differentially excreted proteins (Figure 3), that were associated with pathogenic status, such as glomerular and tubular dysfunction and other types of diseases. Accordingly, these proteins were classified into categories of pathogenesis in Table 2.

In the 3 biological replicates, transferrin (*TF*), ceruloplasmin precursor (*CP*), mannose-binding lectin-associated serine protease-2 precursor (*MASP2*), alpha-1-antitrypsin (*A1AT*), haptoglobin (*HP*), and basement membrane-specific heparin sulfate proteoglycan core protein (*HSPG*) were associated with glomerular dysfunction; except for *MASP2* and *HSPG*, all were upregulated in microalbuminuric versus normoalbuminuric urinary proteomes (Table 2).

Moreover, several differentially excreted proteins that were related to tubular dysfunction, such as vitamin D-binding protein (*VDBP*) and alpha-1-acid glycoprotein 1 precursor (*AGP1*) were selected for further validation. *VDBP* and *AGP1* were upregulated in microalbuminuria versus normoalbuminuria (Table 2).

In addition, *FABP* (fatty acid-binding protein) and *PSCA* (prostate stem cell antigen) correlate with other types of disease, and *PSCA* was selected for further validation. In this iTRAQ experiment, *FABP* was downregulated, whereas *PSCA* expression increased in the microalbuminuric versus normoalbuminuric urinary proteome (Table 2).

### 3.6. Identification of Differentially Excreted Proteins Using 2-D Gel Electrophoresis

**Figure 5:**
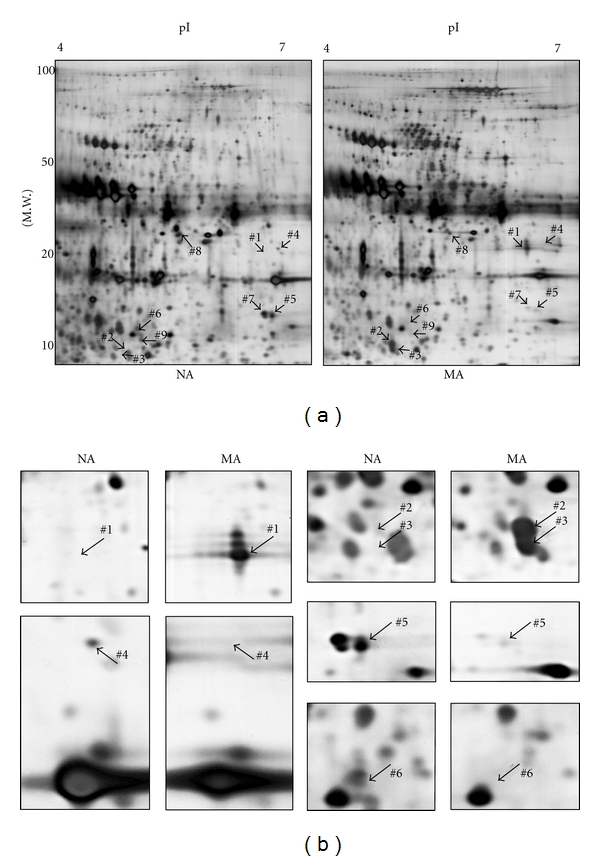
Differentially excreted proteins by 2-DE in microalbuminuria versus normoalbuminuria. (a) Representative whole 2-DE images of normoalbuminuric (NA) and microalbuminuric (MA) urine. Total protein (100 *μ*g) samples were loaded onto IPG strips (pH 4–7, nonlinear) for IEF and separated in the second dimension on a 12% polyacrylamide gel. The horizontal and vertical axes represent p*I* and molecular weight, respectively. The arrowed numbers denote for differentially excreted proteins and correspond to the proteins in Table 3. (b) Magnified sections of differentially excreted proteins in the 2-DE gel. Some arrowed proteins in the 2-DE gel (Figure 5(a)) were magnified side by side to compare their relative expression.

Differential protein expression between microalbuminuric and normoalbuminuric urine was also measured using the 2-D gel electrophoresis in pooled NA4 and MA4 urine (Figure 1 and Table 1). In triplicate 2-DE analysis (Figures 5(a) and 5(b)), two proteins (regulator of telomere elongation helicase 1: *RTEL1* and serum albumin: *ALB*) were upregulated, and 5 proteins (basement membrane-specific heparan sulfate proteoglycan core protein: *HSPG*, fatty acid-binding protein: *FABP*, mannose binding lectin-associated serine protease-2: *MASP2*, AMBP protein: *AMBP*, and Fibulin-5: *FBLN5*) were downregulated in microalbuminuric urine (Figures 5(a) and 5(b)).

 Of the 7 proteins that were identified by 2-DE, 4 had the same pattern of differential excretion as in the iTRAQ experiment. Specifically, the spots that corresponded to serum albumin were upregulated by 2-DE (Figure 3(b), spots 1: 50.8 ± 15.3, 2: 17.5 ± 4.0, and 3: 14.2 ± 2.5) and iTRAQ (iTRAQ: 3.09). In contrast, *HSPG* (spot 4: 0.28 ± 0.041 and iTRAQ: 0.68), *FABP* (spot 5: 0.27 ± 0.037 and iTRAQ: 0.29), and *MASP2* (spot 6: 0.10 ± 0.005 and iTRAQ: 0.29) were significantly downregulated in both techniques (Table 3). *AMBP* (spot 7) was downregulated by 2-DE (0.20 ± 0.002) but upregulated in the iTRAQ experiment (1.44). Two proteins were identified by 2-DE alone—regulator of telomere elongation helicase 1 (spot 8: 3.0 ± 0.5) was upregulated and fibulin-5 precursor (spot 9: 0.12 ± 0.007) was downregulated (Table 3).

### 3.7. Validation of Differentially Expressed Proteins from iTRAQ by Western Blot

**Figure 6:**
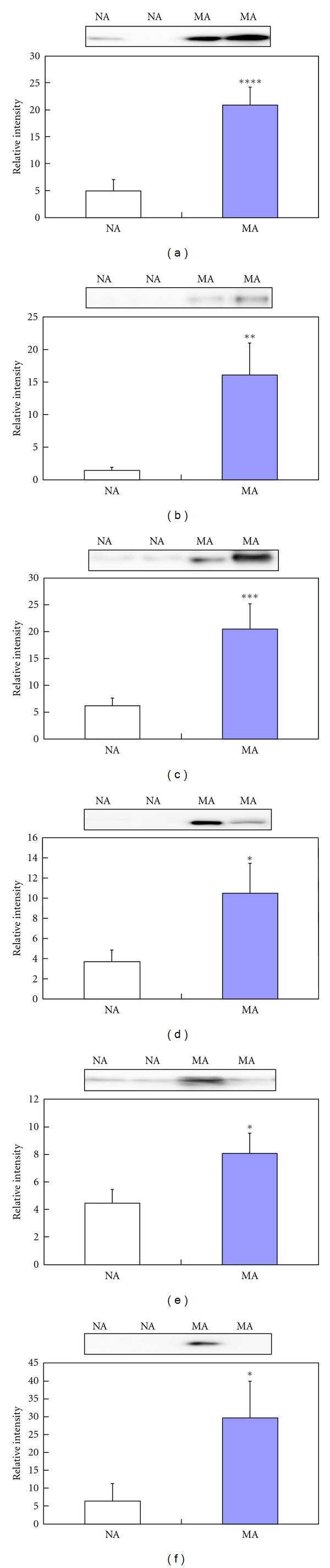
Validation using Western blot for six representative differentially excreted proteins. The concentrations of transferrin (a), ceruloplasmin (b), *α*1-antitrypsin (c), vitamin D-binding protein (d), *α*1-acid glycoprotein (e), and haptoglobin (f) are significantly higher in microalbuminuric patients versus normoalbuminuric urine. The relative intensities on the vertical axis indicate normalized values versus the representative control. Each bar represents the mean ± SEM, based on the relative intensities of the gel bands. Statistical significance for the differences (*P < 0.05; **P < 0.01; ***P < 0.005; ****P < 0.0005) were determined by paired Student's *t*-test. Two representative NA and MA blots are shown at the top of the bar graphs.

To validate the differentially excreted proteins from the iTRAQ results, 6 proteins (*TF*, *CP*, *A1AT*, *VDBP*, *AGP1*, and *HP*) that were associated with pathogenic status were subjected to Western blot. The Western blot results were consistent with the iTRAQ findings (Figure 6 and Table 2): *TF* (4.66 ± 1.41 and *P* < 0.0005), *CP* (11.16 ± 0.38 and *P* < 0.01), *A1AT* (3.36 ± 0.03 and *P* < 0.005), *VDBP* (2.88 ± 0.11 and *P* < 0.05), *AGP1* (1.82 ± 0.08 and *P* < 0.05), and *HP* (7.28 ± 5.52 and *P* < 0.05) were upregulated in microalbumiuric versus normoalbuminuric urine.

### 3.8. MRM Validation for 7 Selected Biomarker Candidates

To verify the 7 biomarker candidates (*TF*, *CP*, *A1AT*, *HP*, *VDBP*, *AGP1*, and *PSCA*), MRM was performed using 9 individual normoalbuminuric and 14 microalbuminuric samples (Figure 1 and Table 1). The peak area for each Q1/Q3 transition (Table 4) for the candidates was first normalized to the peak area of beta-galactosidase that was spiked with 50 fmol as the internal standard and compared between microalbuminuric versus normoalbuminuric samples.

**Figure 7:**
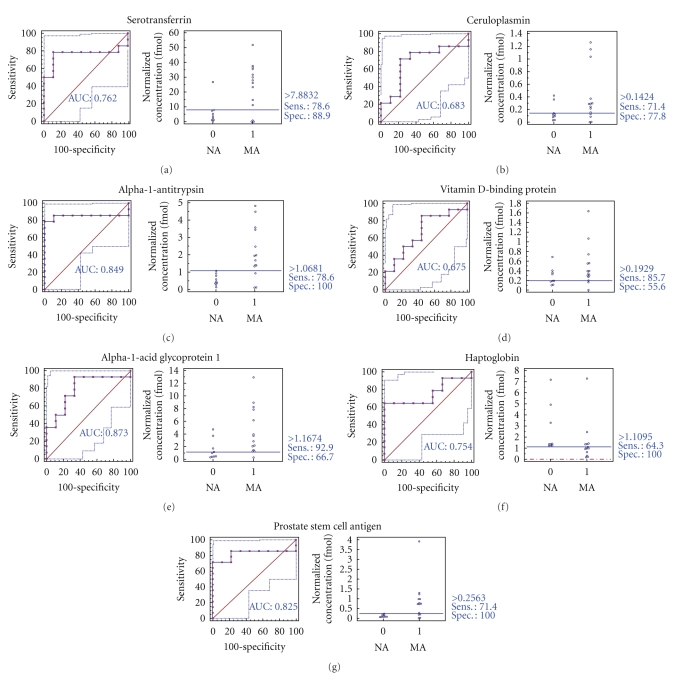
ROC curves and interactive plots for MRM validation in normoalbuminuric versus microalbuminuric urine. Seven biomarker candidates (*TF*, *CP*, *A1AT*, *VDBP*, *AGP1*, *HP*, and *PSCA*) were validated by MRM, in which 9 normoalbuminuric and 14 microalbuminuric urine samples were used. (a)–(g) Interactive plots and ROC curves for *TF*, *CP*, *A1AT*, *VDBP*, *AGP1*, *HP*, and *PSCA*. In the ROC curves, the solid lines represent the corresponding score in sensitivity (*x*-axis) and 100-specificity (*y*-axis). In the interactive plots, the *y*-axis indicates the normalized concentration of the target protein against the spiked internal standard (50 fmol of beta-galactosidase peptide). Sens. and Spec. represent the sensitivity and specificity for the target proteins, respectively. The AUC values are shown inside the ROC curves.

**Figure 8:**
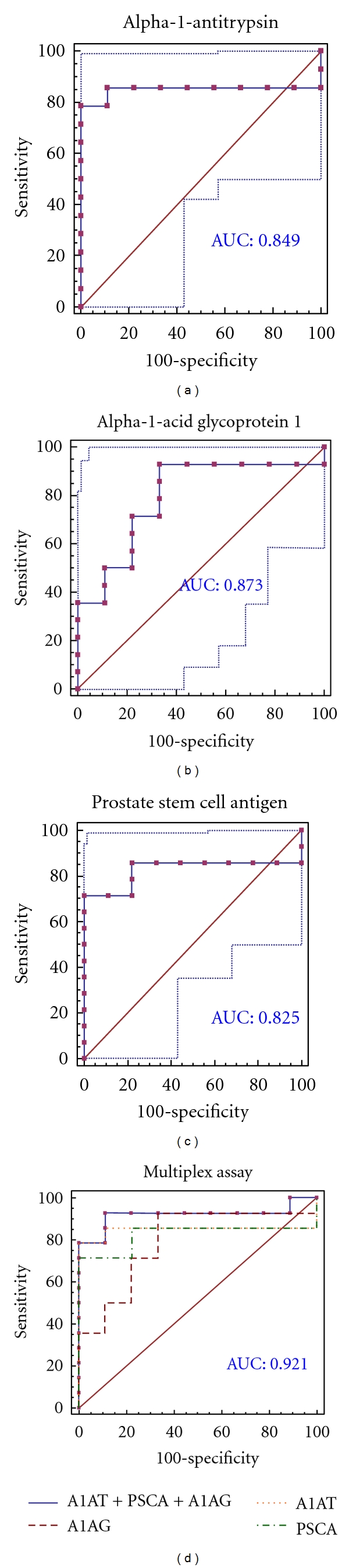
ROC curves for three candidate biomarkers and the 3-marker panel. MRM validation was performed for (a) alpha-1-antitrypsin, (b) alpha-1-acid glycoprotein 1, (c) prostate stem cell antigen, and (d) their combination, generating AUC values of 0.849, 0.873, and 0.825, respectively, whereas the combination resulted in a merged AUC of 0.921.

MRM validation was assessed by interactive plots and ROC curves, represented by the peak area of each Q1/Q3 transition.  Figure 7 shows the interactive plots and ROC curves for *TF*, *CP*, *A1AT*, *VDBP*, *AGP1*, *HP*, and *PSCA* with regard to sensitivity, specificity, and relative concentrations versus beta-galactosidase. In the ROC curves, *TF*, *A1AT*, *AGP1*, *HP*, and *PSCA* had excellent area under the curve (AUC) values (0.762, 0.849, 0.873, 0.754, and 0.825, resp.), as did *CP* and *VDBP*, to a lesser extent (0.683 and 0.675, resp.) (Figure 7). Particularly, the merged ROC curve combining 3 biomarker candidates (alpha-1-antitrypsin, alpha-1-acid glycoprotein 1, and prostate stem cell antigen) resulted in the improved AUC value of 0.921, which is greater than those of the individual proteins (0.849, 0.873, and 0.825 for alpha-1-antitrypsin, alpha-1-acid glycoprotein 1, and prostate stem cell antigen, resp.) (Figure 8).

In the interactive plots, *TF*, *CP*, *A1AT*, *VDBP*, *AGP1*, and *PSCA* were upregulated in microalbuminuric versus normoalbuminuric urine, whereas *HP* was down-regulated. *TF*, *CP*, *A1AT*, *VDBP*, *AGP1*, and *PSCA* had the same excretion patterns by iTRAQ and western blot; conversely, *HP* had the opposite excretion pattern.

## 4. Discussion

### 4.1. Differentially Excreted Proteomes between Microalbuminuria and Normoalbuminuria

To identify and quantify proteins that were associated with diabetic nephropathy in microalbuminuric and normoalbuminuric urine, we used relative quantitative proteomic techniques, such as iTRAQ, 2-DE, Western blot, and MRM. In our iTRAQ experiment, 710 urinary proteins were identified at a >95% confidence level, of which 196 were differentially excreted by >1.25 or <0.80—99 and 97 proteins were up- and down-regulated, respectively (Appendix A).

Recently, the Urinary Protein Biomarker (UPB) database was constructed and published, in which 205 publications were curated manually [29]. Using this database, we can easily determine whether a biomarker candidate has been identified by another group for the same disease and evaluate its disease specificity. Thirty-six of the 196 quantified proteins from our iTRAQ experiment were registered in the UPB database; the remaining 160 proteins were not listed. Subsequently, 196 differentially excreted proteins yielded 10 preliminary biomarker candidates for further validation.

The “molecular function” subcategories accounted for 196 differentially excreted proteins, of which “immunity” represented 25 of the quantitated proteins—the largest component (Figure 3). This proportion reflects the increased inflammatory reactions and higher vascular lesion counts in kidneys during the development of diabetic nephropathy. Through detailed association searches between diabetic nephropathy and the 196 differentially excreted proteins using relevant databases and references, we identified and classified several biomarker candidates that were associated with pathogenic status, such as glomerular and tubular dysfunction and other types of disease (Table 2).

Consequently, 10 proteins were selected for preliminary validation studies such as 2-DE, Western blot, and MRM. For example, 3 proteins (*HSPG*, *FABP*, and *MASP2*) that were identified by 2-DE had the same pattern of differential excretion in the 2-DE and iTRAQ experiments (Table 2). Six differentially excreted proteins (*TF*, *CP*, *A1AT*, *VDBP*, *AGP1*, and *HP*) were validated by Western blot, for which the patterns of excretion were consistent with the iTRAQ results (Table 2).

Recently, an optimized quantitative proteomic strategy in urinary proteomic analysis was proposed for urine biomarker discovery using a small set of samples [21]. According to this report, the initial amount of proteins that is analyzed and the precipitation method (methanol precipitation) of the urine proteins are critical. Notably, our preparation methods for urinary proteomes approximated this optimized protocol, although we used acetone precipitation instead of methanol precipitation.

Nevertheless, an advantage of our study was that we used a large collection of urine samples from 86 diabetic patients to perform 3 iTRAQ experiments, including 3 biological replicates and 2 technical replicates, resulting in more reliable statistical significance.

### 4.2. Differentially Excreted Proteome and Glomerular Dysfunction

 Glomerular dysfunction is caused by GBM thickening and mesangial expansion due to ECM accumulation [2], and several proteins, such as *TF*, *CP*, *MASP2*, *A1AT*, *HP*, and *HSPG*, were associated with glomerular dysfunction.

Transferrin-to-creatinine and ceruloplasmin-to-creatinine ratios are known to reflect changes in renal hemodynamics, and these ratios are significantly higher microalbuminuric patient than normoalbuminuric patients [30]. This result is caused by elevated intraglomerular hydraulic pressure, which leads to the development of diabetic glomerulosclerosis [30–32]. Moreover, *TF* and *CP* were listed in the UPB database, showing upregulation. *TF* is associated with diabetic nephropathy, normoalbuminuric type 2 diabetes, kidney calculi, and ureteropelvic junction obstruction, whereas *CP* is linked to diabetic nephropathy and normoalbuminuric type 2 diabetes. In our iTRAQ and 2-DE, *TF* and *CP* urinary proteins were commonly upregulated in the comparison of microalbuminuric and normoalbuminuric urinary proteome (Table 2).

*MASP2* is a serum protease that activates the complement cascade, which regulates the maintenance of glomerular permeability and the pathogenesis of focal segmental glomerulosclerosis [33]. *MASP2* was not listed in the UPB database, and in this iTRAQ and 2-DE, this protein was commonly downregulated in comparison of microalbuminuric and normoalbuminuric urinary proteome (Table 2).

*A1AT* is a serine protease inhibitor, which prevents neutrophil elastase by degrading ECM proteins, which maintains vascular elasticity and glomerular integrity [13, 34]. In a previous study, *A1AT* excretion was elevated in microalbuminuria, which can cause matrix molecules to accumulate [35]. *A1AT* was also listed in the UPB database, showing upregulation; it is associated with diabetic nephropathy, severe acute pancreatitis, kidney calculi, nephrotic syndrome, and ureteropelvic junction obstruction. In our iTRAQ and Western blot, *A1AT* was also upregulated in the comparison of microalbuminuric and normoalbuminuric urinary proteome (Table 2).

Haptoglobin (*HP*) is an acute phase protein that binds to free hemoglobin (Hb) with the highest affinity. Formation of the Hb-HP complex prevents the loss of renal iron and oxidative damage that are driven by free Hb. Specifically, *HP* mediates complement-dependent podocyte damage [36]. Thus, complement activation results in the release of proteases, oxidants, and growth factors, damaging the functional integrity of the GBM. *HP*, which was downregulated and is associated with diabetic nephropathy and type 2 diabetes mellitus, appeared in the UPB database. However, in our iTRAQ and Western blot, *HP* was commonly upregulated in the comparison of microalbuminuric and normoalbuminuric urinary proteome (Table 2).

*HSPG* is present in the basement membrane of every vascularized organ, including the GBM. The highly negatively charged side chains of *HSPG* are important determinants for the charge-selective permeability of the GBM [37]. Under hyperglycemic conditions, the loss of *HSPG* from the GBM alters the charge-selective properties of the glomerular capillary, causing increased filtration of negatively charged albumin [37, 38]. *HSPG* is not registered in the UPB database, and in our iTRAQ and 2-DE, *HSPG* was commonly downregulated in the comparison of microalbuminuric and normoalbuminuric urinary proteome (Table 2).

### 4.3. Differentially Excreted Proteome and Tubular Dysfunction and Other Types of Diseases

Low-molecular-weight proteins, such as *VDBP* and *AGP1*, are associated with kidney tubular dysfunction and were commonly upregulated in the iTRAQ and Western blot (Table 2). Unlike high-molecular-weight proteins, they are filtered in the glomerulus on the basis of charge selectivity and pore size of the GBM and are reabsorbed into proximal renal tubules under normal conditions [38]. If reabsorption is impaired, however, these proteins can be overexcreted into urine.

*VDBP* is a multifunctional serum glycoprotein and is an important mediator in immunopathogenesis because it is associated with immunoglobulin receptors on the surfaces of B- and T-lymphocytes [12]. Moreover, *VDBP* binds to circulating vitamin D metabolites with high affinity [39], and *VDBP*-bound 25-hydroxyvitamin D3 crosses the GBM and is reabsorbed by proximal tubular cells in a megalin-dependent manner, suggesting that it controls renal uptake and the activation of metabolites [4, 40]. *VDBP*, also listed in the UPB database, was upregulated and is associated with diabetic nephropathy and Dents disease. In our iTRAQ and Western blot, *VDBP* was commonly upregulated in the comparison of microalbuminuric and normoalbuminuric urinary proteome (Table 2).

*AGP1* is synthesized in response to systemic tissue injury, inflammation, and infection, like most other acute phase proteins [7, 41]. Further, increased levels of *AGP1* reflect elevated levels of cytokines, such as interleukin-1 (IL-1), IL-6, and tumor necrosis factor alpha (TNF-*α*), which are associated with type 2 diabetes [7]. A study has demonstrated that *AGP1* is an indicator of tubular disorder in multiple myeloma [42], and another has shown that serum and urinary *AGP1* levels are elevated in type 2 diabetic patients with kidney disorders [5, 7]. *AGP1 *was listed in the UPB database, showing upregulation, and is associated with diabetic nephropathy, diabetic kidney disorder, preeclampsia, and acute appendicitis. In our iTRAQ and Western blot, *AGP1* was also commonly upregulated in the comparison of microalbuminuric and normoalbuminuric urinary proteome (Table 2).

*FABP* (fatty acid-binding protein: *FABP*) correlated with other types of diseases [43] and was also chosen for further validation. *FABP *was not listed in the UPB database, and in this iTRAQ and 2-DE, *FABP* was commonly downregulated in the comparison of microalbuminuric and normoalbuminuric urinary proteome (Table 2).

*PSCA* is highly expressed in the prostate and, to a lesser extent, in the bladder, placenta, colon, kidney, and stomach. Moreover, it is upregulated in prostate cancer and is detected in cancers of the bladder and pancreas [44]. *PSCA* was not listed in the UPB database but was upregulated in the microalbuminuric versus normoalbuminuric urinary proteome in our iTRAQ experiment (Table 2). No relationship between prostate stem cell antigen and diabetic nephropathy has been reported.

### 4.4. Validation of Differentially Excreted Proteins Using MRM

 For the MRM experiments, we used a bacterial beta galactosidase peptide as the internal standard for relative quatitation [28]. Seven preliminary biomarker candidates (*TF*, *CP*, *A1AT*, *VDBP*, *AGP1*, *HP*, and *PSCA*) were confirmed in 9 normoalbuminuric and 14 microalbuminuric urine samples by MRM (Figure 7).

In the interactive plots, *TF*, *CP*, *A1AT*, *VDBP*, *AGP1*, and *PSCA *were preferentially excreted in microalbuminuria versus normoalbuminuria, whereas *HP* was downregulated. *TF*, *CP*, *A1AT*, *VDBP*, and *AGP1* had the same pattern of excretion in the iTRAQ and western analysis, and *HP* had the opposite pattern between the MRM and Western analysis. *HP* consists of *α*-chain (amino acid sequence: 19–160) and *β*-chain (aminoacid sequence: 162–406), which are connected by disulfide bridges. In the MRM experiment, the transition (amino acid sequence: 203–215) in the *β*-chains was used for the relative quantitation of *HP*. In contrast, portions of both the *α*-chain and *β*-chain were used in the iTRAQ quantitation (sequence coverage: 31.0%), and an antibody that targeted a sequence in the *α*-chain was used for the Western blot analysis. It is conceivable that these disparate targets resulted in contradictory patterns between iTRAQ, Western blot, and MRM. Regardless of the methods or targets, reproducible patterns must be obtained with each method.

Furthermore, we performed a multiplex assay to improve AUC values with 3 biomarker candidates (alpha-1-antitrypsin, alpha-1-acid glycoprotein 1, and prostate stem cell antigen), obtaining a merged AUC value of 0.921, which is greater than those of the individual proteins (0.849, 0.873, and 0.825 for alpha-1-antitrypsin, alpha-1-acid glycoprotein 1, and prostate stem cell antigen, resp.) (Figure 8).

Although our results require further validation in a larger collection of urine samples that contains various control samples, it appears that *A1AT*, *AGP1*, and *PSCA* are excellent biomarker candidates, with AUC values > 0.8; combining the candidates improved the AUC value of 0.921. Accordingly, the other differentially expressed proteins from our iTRAQ experiment in Appendix A are potential candidates for further validation in obtaining DN biomarkers.

## 5. Conclusions

Microalbuminuria is used as a noninvasive index for the detection of diabetic renal disease. Yet, more specific and accurate biomarkers for DN are required, particularly in type 2 diabetic patients, due to several reasons, including nonspecific detection in nondiabetic renal disease, cardiovascular disease, inflammation, and hypertension. In our iTRAQ experiments, 710 urinary proteins were identified at a >95% confidence level, of which 196 were differentially excreted by >1.25 or <0.80—99 and 97 proteins were up- and down-regulated, respectively (Appendix A).

We prioritized 196 proteins to select preliminary biomarker candidates by characterizing them with regard to “biological process” and “molecular function” and associating them with pathogenesis. Consequently, 10 proteins were selected. To confirm and validate these candidates, 2-DE, Western blot, and MRM were performed. Based on the MRM results, *A1AT*, *AGP1*, and *PSCA*, which had AUC values > 0.8, are good biomarker candidates, and we improved the AUC value to 0.921 on combining the 3 proteins.

Further validation studies on other differentially excreted proteins might contribute to a greater understanding of the mechanism of renal dysfunction and its association with the pathogenesis of DN, facilitating the development of better biomarkers for DN.

## Figures and Tables

**Figure 9:**
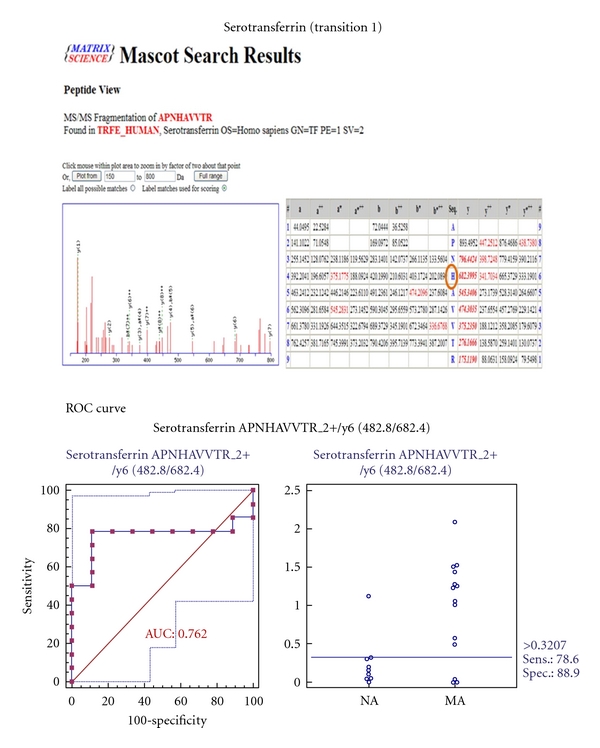


**Figure 10:**
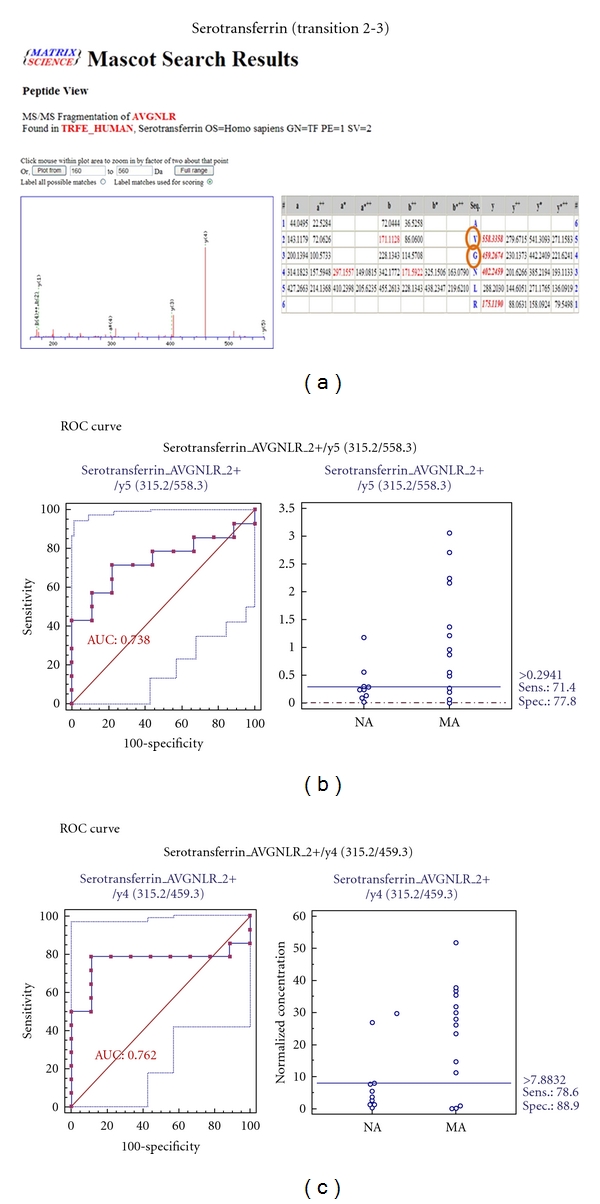


**Figure 11:**
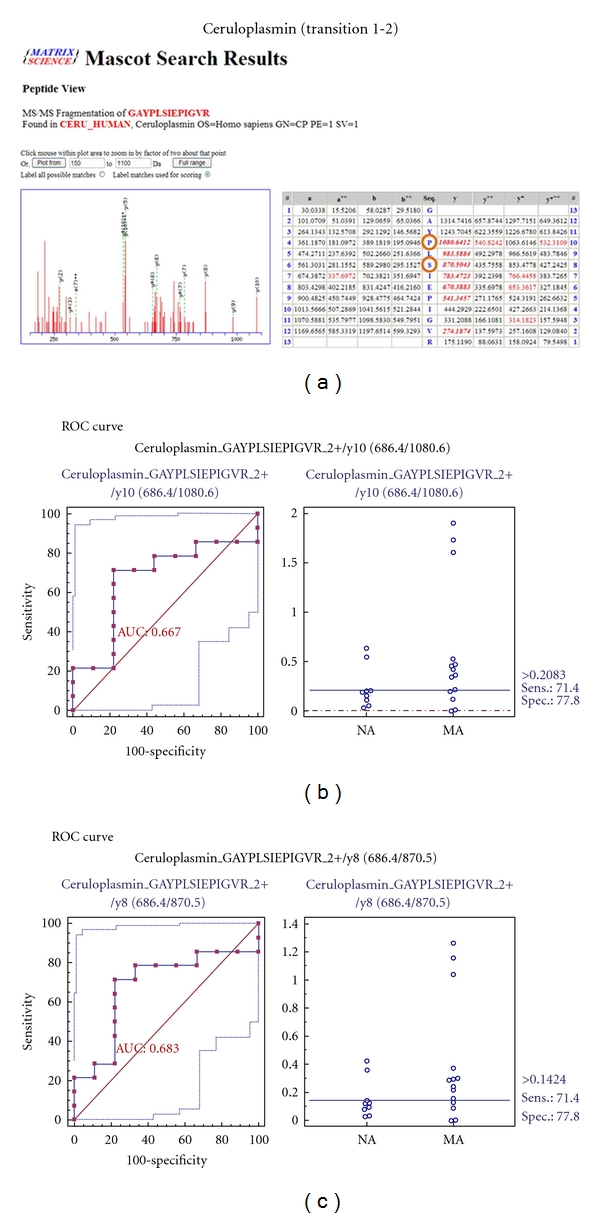


**Figure 12:**
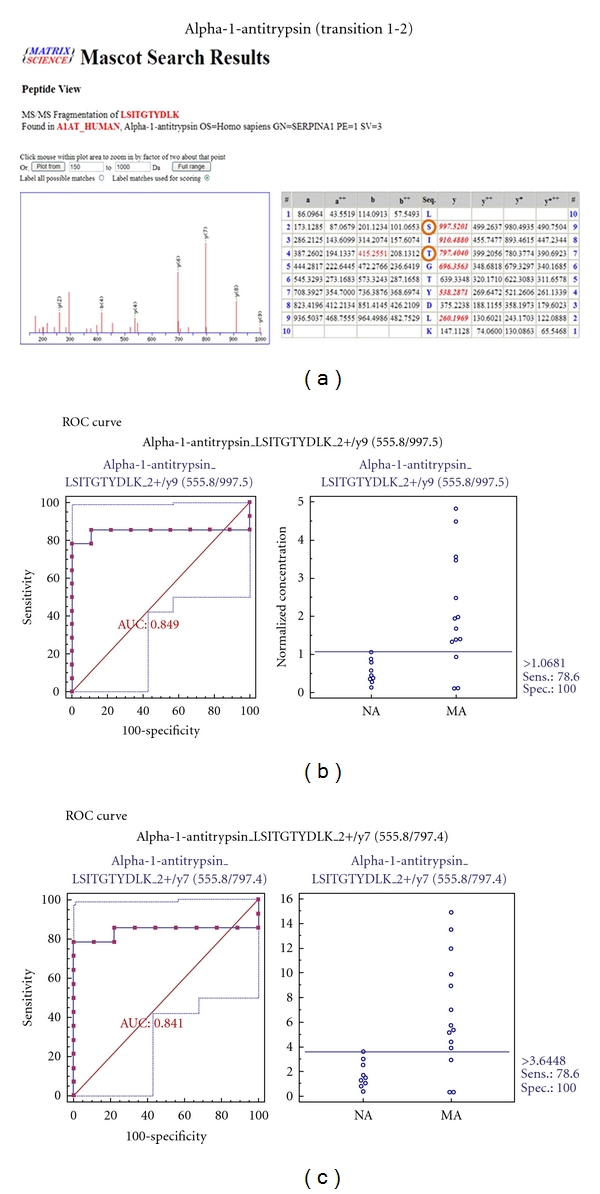


**Figure 13:**
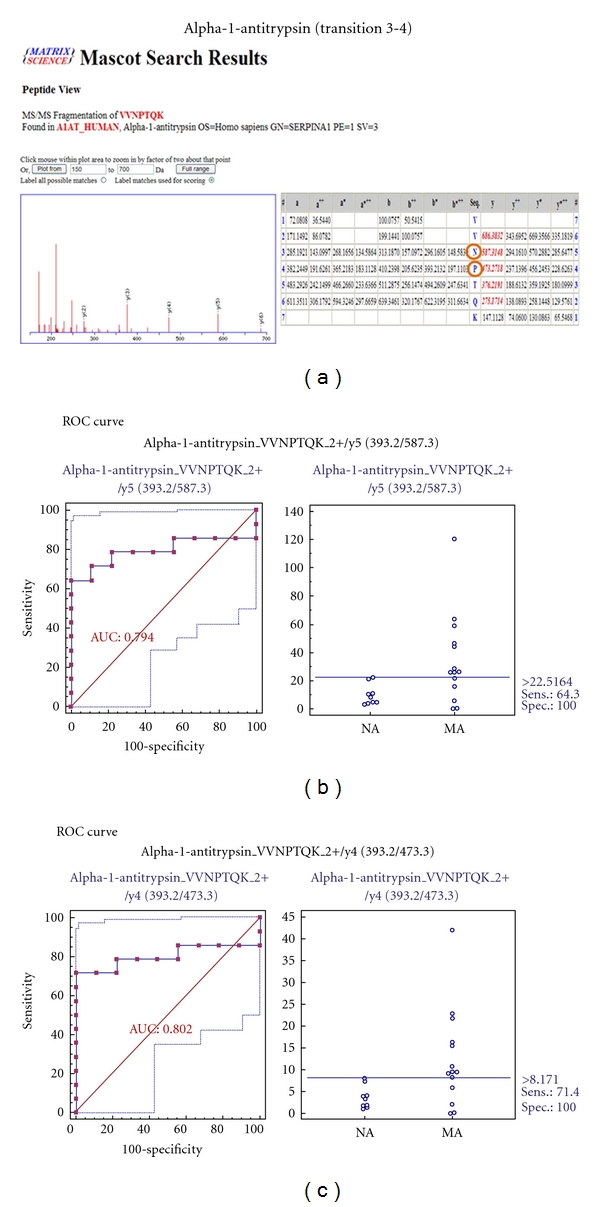


**Figure 14:**
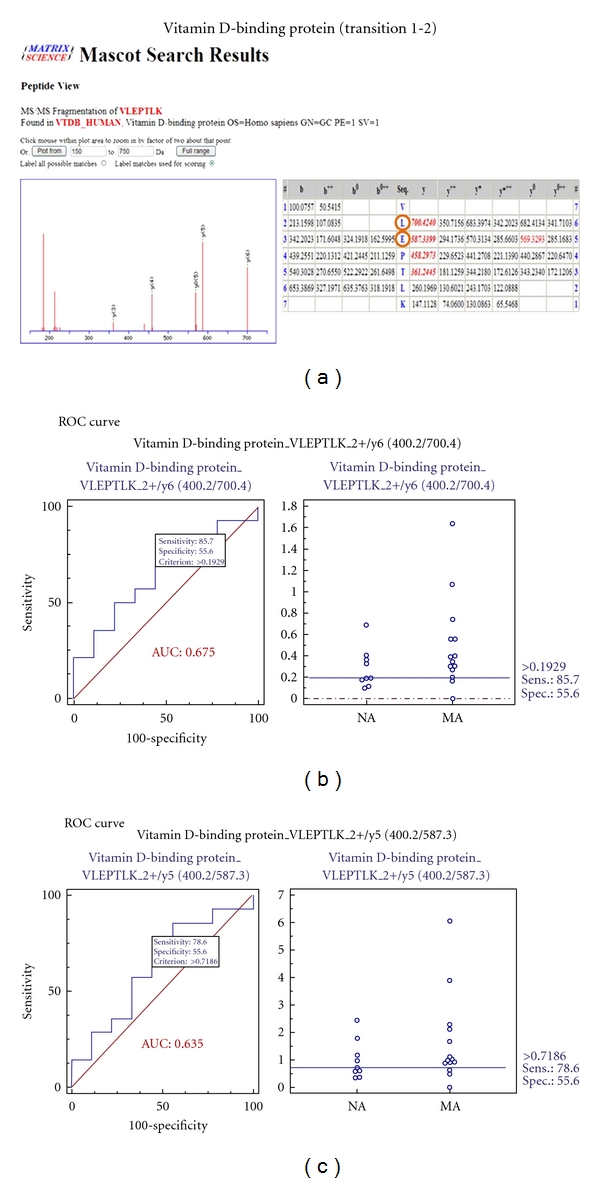


**Figure 15:**
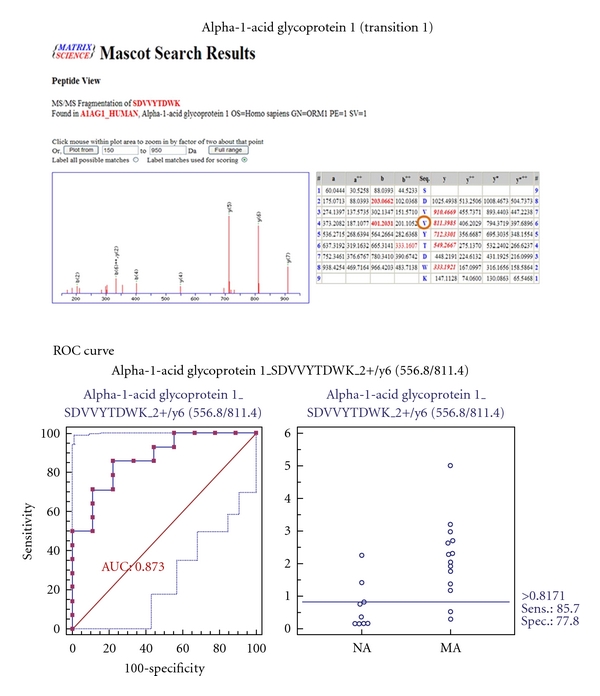


**Figure 16:**
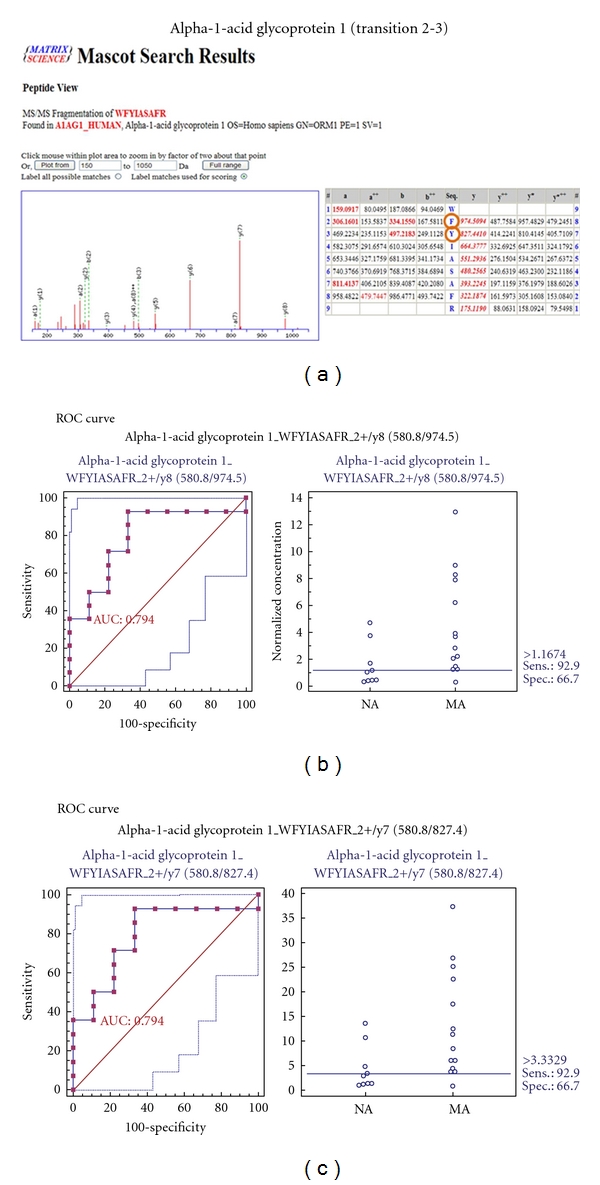


**Figure 17:**
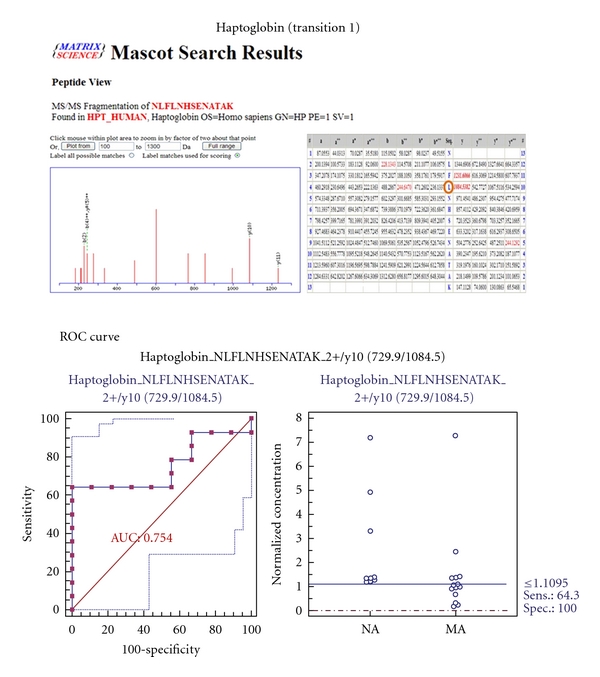


**Figure 18:**
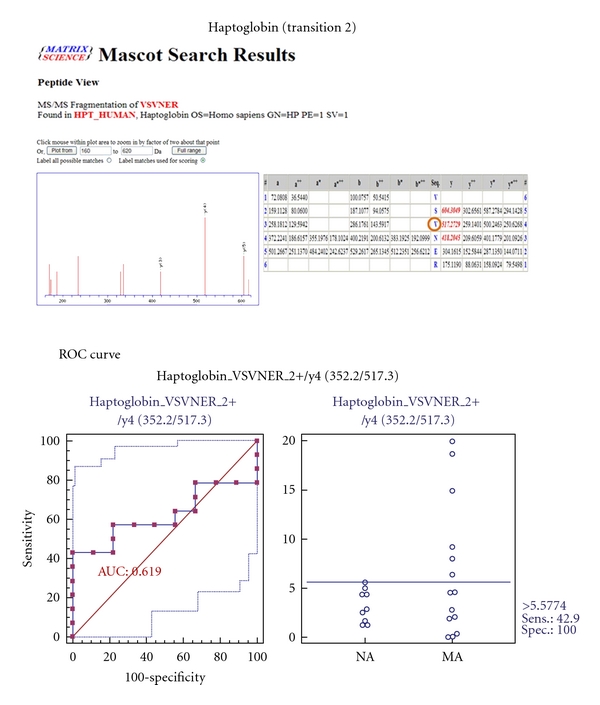


**Figure 19:**
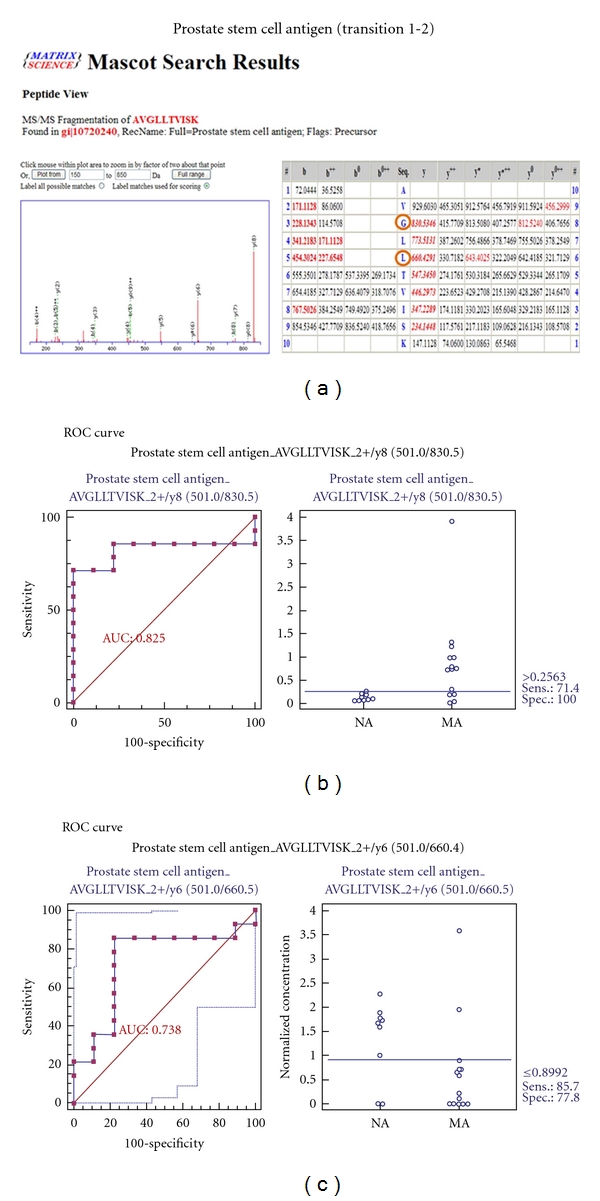


**Table 1:** Clinical characteristics of normoalbuminuric (NA) and microalbuminuric (MA) type 2 diabetic patients.

Characteristics	NA 1^a^	MA 1^a^	NA 2^b^	MA 2^b^	NA 3^c^	MA 3^c^	NA 4^d^	MA 4^d^
	(*n* = 9)	(*n* = 9)	(*n* = 9)	(*n* = 9)	(*n* = 9)	(*n* = 9)	(*n* = 16)	(*n* = 16)
Gender (M/F)	4/5	5/4	5/4	4/5	5/4	4/5	9/7	9/7
Mean age (years)	62.4 ± 8.0 (49–72)	66.4 ± 7.8 (55–82)	60.7 ± 4.7 (54–67)	61.9 ± 2.7 (56–65)	64.9 ± 5.3 (56–73)	62.3 ± 4.4 (41–72)	63.7 ± 7.5 (49–72)	63.2 ± 9.8 (44–82)
Duration of diabetes (years)	9.1 ± 4.4	10.4 ± 7.0	8.7 ± 5.0	7.3 ± 4.7	13.0 ± 9.0	10.6 ± 7.7	9.9 ± 4.8	11.6 ± 7.3
BMI (kg/m^2^)	25.4 ± 3.0	24.9 ± 3.3	23.8 ± 2.1	24.9 ± 3.6	23.1 ± 2.7	22.6 ± 2.6	24.4 ± 2.9	25.1 ± 2.9
Fasting plasma glucose (mg/dL)	130.8 ± 21.1	133.8 ± 36.4	131.3 ± 31.9	135.8 ± 41.3	117.8 ± 21.7	117.6 ± 26.6	132.4 ± 19.2	144.8 ± 35.7
HbA1C (%)	6.8 ± 0.7	6.8 ± 0.9	6.9 ± 0.7	7.4 ± 0.8	7.2 ± 0.7	7.2 ± 0.7	6.8 ± 0.6	7.0 ± 0.8
Blood urea nitrogen (mg/dL)	15.1 ± 4.8	17.1 ± 4.9	13.8 ± 2.4	15.7 ± 3.9	16.8 ± 3.4	17.7 ± 4.4	15.1 ± 3.9	16.4 ± 4.1
Serum creatinine (mg/dL)	1.0 ± 0.1	1.1 ± 0.2	0.9 ± 0.2	1.0 ± 0.1	1.0 ± 0.2	1.0 ± 0.2	0.98 ± 0.13	1.0 ± 0.15
Serum total cholesterol (mg/dL)	184.4 ± 34.5	180.6 ± 29.1	164.2 ± 25.5	165.6 ± 24.5	164.9 ± 22.2	175.4 ± 50.7	181.5 ± 29.7	182.1 ± 25.5
Serum HDL cholesterol (mg/dL)	48.1 ± 12.2	46.1 ± 8.8	52.3 ± 9.5	48.9 ± 8.3	55.0 ± 7.8	41.5 ± 4.7	46.4 ± 10.8	46.8 ± 7.7
Serum LDL cholesterol (mg/dL)	99.8 ± 26.6	102.6 ± 22.3	91.0 ± 21.6	92.0 ± 27.6	85.9 ± 20.3	98.8 ± 30.0	102.0 ± 23.4	104.1 ± 19.7
Serum triglycerides (mg/dL)	119.9 ± 37.0	158.6 ± 55.9	132.9 ± 66.1	177 ± 134.7	128.3 ± 82.8	186.3 ± 66.8	126.5 ± 56.5	145.4 ± 51.6
Albumin : creatinine ratio (mg/g)	12.2 ± 7.1	120.5 ± 70.7^e^	10.0 ± 3.6	82.6 ± 41.9^f^	8.8 ± 1.6	86.1 ± 47.1^g^	9.8 ± 6.9	107.4 ± 69.4 ^h^
Pooled urine concentration (mg/mL)	3.7 ± 1.6	6.9 ± 3.4	4.1 ± 1.2	8.1 ± 4.2	4.7 ± 1.2	7.6 ± 3.8	4.3 ± 1.8	7.1 ± 4.5

^a–c^Sample sets for the three iTRAQ experiments, ^d^sample sets for 2-DE, ^ e^ *P* < 0.001 for NA1 versus MA1, ^ f^ *P* < 0.001 for NA2 versus MA2, ^g^ *P* < 0.05 for NA3 versus MA3, and ^h^ *P* < 0.001 for NA4 versus MA4. Data are expressed as the mean ± SD.

**Table 2:** Selected 10 differentially excreted proteins related to pathogenic status in microalbumiuric versus normoalbuminuric urines.

Pathogenic status	*N*	Number of unique peptides^a^	Accession number^b^	Gene name^c^	MA : NA expression		
					iTRAQ^d^	2-DE^e^	WB^f^
Glomerular dysfunction	1	15	spt∣P02787	*TF*	1.86	—	4.66 ± 1.41
	2	47	Spt∣P00450	*CP*	2.09	—	11.16 ± 0.38
	3	209	spt∣P01009	*A1AT*	1.42	—	3.36 ± 0.03
	4	49	spt∣P00738	*HP*	2.35	—	7.28 ± 5.52
	5	10	trm∣Q9UMV3	*MASP2*	0.29	0.10 ± 0.005	—
	6	235	spt∣P98160	*HSPG*	0.68	0.28 ± 0.041	—

Tubular dysfunction	7	9	spt∣P02774	*GC*, *VDBP *	2.44	—	2.88 ± 0.11
	8	414	spt∣P02763	*ORM1*, *AGP1 *	2.04	—	1.82 ± 0.08

Other types of diseases	9	9	spt∣Q01469	*FABP*	0.29	0.27 ± 0.037	—
	10	5	trm∣O43653	*PSCA*	1.70	—	—

^a^The numbers of unique peptides and MS/MS spectrum observed by ProteinPilot software were determined only for those peptides with ≥95% confidence. ^b^Accession numbers represent entries in the Human CDS database (human KBMS 5.0, 2005-03-02; a total of 187,748 entries provided by Applied Biosystems). ^c^Gene name from the Expasy database correspond to protein accession number ^b^from the Human CDS database (human KBMS 5.0, 2005-03-02; a total of 187,748 entries provided by Applied Biosystems). ^d–f^Ratio of differentially excreted protein for iTRAQ, 2-DE and WB in microalbumiuric versus normoalbuminuric urines, respectively.

**Table 3:** Differentially expressed proteins by 2-DE in microalbuminuria versus normoalbuminuria.

Gene name^a^	Accession number^b^	Up-/down-regulated	Mol. Mass, Da (pI)^c^	Peptides matched	Total ion C.I.%^d^	Ma/Na (2-DE)^f^	Ma/Na (iTRAQ)^g^
*ALB*	P02768	Up	71317.2 (5.92)	2	100.00	50.8 ± 15.3	3.09
*ALB*	P02768	Up	71317.2 (5.92)	2	99.84	17.5 ± 4.0	3.09
*ALB*	P02768	Up	71317.2 (5.92)	2	100.00	14.2 ± 2.5	3.09
*HSPG*	P98160	Down	468527.5 (6.06)	2	100.00	0.28 ± 0.041	0.68
*FABP*	Q01469	Down	15496.7 (6.6)	1	97.76	0.27 ± 0.037	0.29
*MASP2*	Q9UMV3	Down	75684.6 (5.47)	2	100.00	0.10 ± 0.005	0.29
*AMBP*	P02760	Down	38974 (5.95)	2	99.91	0.20 ± 0.002	1.44
*RTEL1*	Q9NZ71	Up	152278.2 (8.68)	1	96.77	3.0 ± 0.5	—
*FBLN5*	Q9UBX5	Down	50146.7 (4.58)	1	99.36	0.12 ± 0.007	—

^a-b^Gene name from the Expasy database correspond to protein accession number. ^b^Accession numbers represent entries in the Human CDS database. ^c^Molecular mass (mol. mass) is presented by Da, while isoelectric point stands for pI. ^d^Total ion score and total ion CI % for MALDI-TOF/TOF were calculated using GPS v3.5 in the MASCOT search program (v2.0). ^f-g^Ratio of differentially excreted protein for 2-DE and iTRAQ in microalbumiuric versus normoalbuminuric urines, respectively. Data are expressed as the mean ± SD.

**Table 4:** Parameters of MRM Experiment for seven candidate proteins.

Protein name	Q1^a^	Q3^b^	Sequence^c^	Fragment^d^	Charge^e^	CE^f^
Transferrin	482.8	682.4	APNHAVVTR	y6	2+	26
	315.2	558.3	AVGNLR	y5	2+	19
	315.2	459.3	AVGNLR	y4	2+	19
Ceruloplasmin	686.4	1080.0	GAYPLSIEPIGVR	y10	2+	35
	686.39	870.5	GAYPLSIEPIGVR	y8	2+	35
Alpha-1-antitrypsin	555.81	997.5	LSITGTYDLK	y9	2+	29
	555.81	797.4	LSITGTYDLK	y7	2+	29
	393.2	587.3	VVNPTQK	y5	2+	22
	393.2	473.3	VVNPTQK	y5	2+	22
Haptoglobin precursor	729.8	1084.5	NLFLNHSENATAK	y10	2+	37
	352.2	517.3	VSVNER	y4	2+	20
Vitamin D-binding protein	400.2	700.4	VLEPTLK	y6	2+	26
	400.2	587.3	VLEPTLK	y5	2+	26
Alpha-1-acid glycoprotein 1	556.8	811.4	SDVVYTDWK	y6	2+	29
	580.8	974.5	WFYIASAFR	y8	2+	31
	580.8	827.4	WFYIASAFR	y7	2+	31
Prostate stem cell antigen	501.0	830.5	AVGLLTVISK	y8	2+	30
	501.0	660.4	AVGLLTVISK	y6	2+	30

^a-b^Q1 and Q3 (*m/z*) represent the Q1 and Q3 transitions for proteotypic peptide, respectively. ^c^Sequence represents the sequence of proteotypic peptide for target protein. ^d^Fragment type indicates the ion type of the Q3 transition. ^e^Charge represents the charge state of precursor ion. ^f^CE represents collision energy.

**Table 5:** Differentially excreted urinary proteome in microalbumiuric versus normoalbuminuric urine.

*N*	Unique peptides^a^	Accession number^b^	Protein name	Ratio^c^ MA : NA	Pval^d^ MA : NA	EF^e^ MA : NA
1	651	spt∣P02768	Serum albumin	3.09	0.00	1.04
2	337	gb∣AAF01333.1	Serum albumin	0.36	0.00	1.08
3	669	trm∣Q8N4N0	Alpha-2-glycoprotein 1	1.48	0.00	1.02
4	639	rf∣NP_003352.1	uromodulin	0.72	0.00	1.04
5	269	emb∣CAA42438.1	Zn-alpha2-glycoprotein	1.80	0.00	1.06
6	235	spt∣P98160	HSPG	0.68	0.00	1.04
7	209	spt∣P01009	Alpha-1-antitrypsin	1.42	0.00	1.04
8	414	spt∣P02763	Alpha-1-acid glycoprotein 1	2.04	0.00	1.03
9	265	spt∣P02788	Serotransferrin	2.46	0.00	1.10
10	46	spt∣P02760	AMBP protein	1.44	0.00	1.12
11	300	spt∣P07911	Uromodulin	0.24	0.00	1.08
12	106	prf∣765044A	Ig G1 H Nie	0.61	0.00	1.15
13	223	dbj∣BAC85395.1	Unnamed protein product	1.37	0.00	1.10
14	217	emb∣CAA29229.1	Alpha-1-acid glycoprotein 1	2.29	0.00	1.11
15	107	trm∣Q5VU27	Heparan sulfate proteoglycan 2	2.00	0.00	1.18
16	306	spt∣P07998	Ribonuclease pancreatic	0.80	0.00	1.08
17	62	spt∣P41222	Prostaglandin-H2 D-isomerase	1.40	0.00	1.13
18	47	spt∣P00450	Ceruloplasmin	2.09	0.00	1.12
19	117	prf∣763134A	Ig A1 Bur	1.60	0.02	1.39
20	46	cra∣hCP1909255	Serine proteinase inhibitor	1.52	0.00	1.07
21	90	spt∣P10451	Osteopontin	0.57	0.00	1.43
22	9	spt∣P04746	Pancreatic alpha-amylase	0.41	0.00	1.33
23	39	spt∣P02749	Beta-2-glycoprotein I	1.37	0.00	1.07
24	50	trm∣Q9UII8	E-cadherin	1.36	0.00	1.12
25	34	rf∣NP_006112.2	Keratin 1	0.60	0.00	1.16
26	88	spt∣Q14624	ITIH4	0.78	0.00	1.08
27	21	trm∣Q6N025	FN	1.27	0.01	1.20
28	21	trm∣Q8N175	Keratin 10	0.67	0.00	1.27
29	34	emb∣CAA48671.1	Alpha1-antichymotrypsin	1.64	0.00	1.17
30	50	spt∣P00738	Haptoglobin	2.36	0.01	1.24
31	22	gb∣AAA52014.1	Cholesterol esterase	0.46	0.00	1.10
32	63	spt∣P05451	Lithostathine 1 alpha	1.54	0.00	1.05
33	31	trm∣Q6PAU9	Kininogen 1	0.75	0.00	1.18
34	20	spt∣P04217	Alpha-1B-glycoprotein	1.86	0.00	1.31
35	34	spt∣P05155	Plasma protease C1 inhibitor	0.75	0.00	1.12
36	14	trm∣Q8N473	Alpha 1 type I collagen	0.72	0.00	1.14
37	139	trm∣Q6IB74	ORM2 protein	1.48	0.00	1.23
38	22	spt∣Q8WZ75	Roundabout homolog 4	0.34	0.00	1.23
39	29	spt∣P02791	Hemopexin	1.73	0.01	1.33
40	33	trm∣Q6LBL5	GM2 activator protein	1.45	0.00	1.06
41	19	pdb∣1HP7_A	A Chain A, uncleaved alpha-1-antitrypsin	1.76	0.00	1.18
42	23	spt∣P55290	Cadherin-13	0.76	0.00	1.13
43	21	trm∣Q8IZY7	Poly-Ig receptor	0.63	0.00	1.36
44	25	spt∣P05154	Plasma serine protease inhibitor	0.76	0.00	1.12
45	10	trm∣Q96CZ9	Cadherin 11, type 2, isoform 1 preproprotein	0.44	0.00	1.50
46	16	gb∣AAR84237.2	Truncated epidermal growth factor	0.48	0.00	1.25
47	35	spt∣P24855	Deoxyribonuclease I	0.50	0.00	1.12
48	17	trm∣Q7Z645	Collagen, type VI, alpha 1	0.51	0.00	1.17
49	15	dbj∣BAA19556.1	Immunoglobulin light chain V-J region	1.69	0.01	1.38
50	33	emb∣CAA23842.1	Unnamed protein product	1.43	0.00	1.05
51	32	spt∣P08571	CD14	2.36	0.00	1.62
52	26	trm∣Q6GMX2	Hypothetical protein	0.59	0.01	1.24
53	111	emb∣CAA29873.2	Alpha-1-acid glycoprotein 2	2.25	0.00	1.18
54	7	trm∣Q8WY99	Cathepsin C	1.58	0.00	1.17
55	11	spt∣Q92820	Gamma-glutamyl hydrolase	0.54	0.01	1.33
56	13	spt∣P15586	N-acetylglucosamine-6-sulfatase	1.30	0.02	1.23
57	10	gb∣AAQ88523.1	AQGV3103	0.79	0.05	1.26
58	8	trm∣Q8N2F4	Hypothetical protein PSEC0200	0.71	0.01	1.29
59	20	trm∣Q6MZU6	Hypothetical protein DKFZp686C15213	0.39	0.00	1.28
60	27	trm∣Q6LDS3	APS protein	1.34	0.00	1.08
61	9	pdb∣1L9X_D	Structure Of Gamma-Glutamyl Hydrolase	0.69	0.00	1.12
62	15	spt∣P07339	Cathepsin D	1.38	0.01	1.26
63	10	spt∣P51884	Lumican	0.78	0.01	1.20
64	130	dbj∣BAC85483.1	Unnamed protein product	0.73	0.00	1.14
65	9	pdb∣1ATH_B	B Chain B, Antithrombin Iii	1.29	0.00	1.17
66	15	rf∣NP_001822.2	Clusterin isoform 1	0.63	0.00	1.25
67	23	trm∣Q5VW91	Decay accelerating factor for complement	1.26	0.00	1.09
68	7	spt∣P54802	Alpha-N-acetylglucosaminidase	0.50	0.00	1.25
69	12	spt∣Q16270	IGFBP-7	0.79	0.00	1.14
70	8	trm∣Q5VZE3	Golgi phosphoprotein 2	0.38	0.02	1.46
71	10	spt∣P05543	Thyroxine-binding globulin	1.27	0.00	1.11
72	9	spt∣P02774	Vitamin D-binding protein	2.44	0.00	1.15
73	63	rf∣NP_000573.1	Secreted phosphoprotein 1	0.58	0.00	1.24
74	56	spt∣P02671	Fibrinogen alpha/alpha-E chain	0.67	0.00	1.11
75	10	trm∣Q9UBG3	Tumor-related protein	0.32	0.00	1.67
76	15	trm∣O00391	Quiescin Q6	0.77	0.03	1.25
77	36	trm∣Q5VY30	Retinol binding protein 4, plasma	1.38	0.00	1.04
78	8	rf∣NP_004675.2	SPARC-like 1	0.55	0.03	1.70
79	9	spt∣Q92692	Herpesvirus entry mediator B	0.50	0.00	1.31
80	39	trm∣Q96FE7	HGFL protein	0.73	0.00	1.15
81	6	spt∣P43652	Afamin	4.67	0.00	1.33
82	24	pdb∣1QDD_A	Lithostathine	1.73	0.00	1.20
83	24	spt∣P10153	Nonsecretory ribonuclease	0.83	0.01	1.13
84	8	spt∣P16278	Beta-galactosidase	1.50	0.00	1.20
85	7	trm∣Q5VYK1	Collagen, type XII, alpha 1	0.75	0.00	1.15
86	9	emb∣CAA37914.1	Precursor (AA-19 to 692)	1.91	0.01	1.62
87	15	trm∣Q7Z5L0	Unnamed secretory protein	0.56	0.00	1.31
88	11	spt∣P08236	Beta-glucuronidase	1.33	0.00	1.12
89	13	cra∣hCP51001.2	superoxide dismutase 3	0.45	0.00	1.38
90	17	pir∣S13195	Ganglioside M2 activator protein	1.33	0.00	1.18
91	12	cra∣hCP1858145	Protein C receptor, endothelial	1.42	0.00	1.22
92	12	trm∣Q6IAT8	B2M protein	1.48	0.00	1.08
93	7	trm∣Q9Y5X6	Glutamate carboxypeptidase	1.47	0.00	1.04
94	13	spt∣P06702	Calgranulin B	0.41	0.00	1.36
95	11	emb∣CAB90482.1	Human type XVIII collagen	0.56	0.01	1.48
96	7	trm∣O00533	Neural cell adhesion molecule	0.73	0.02	1.29
97	72	spt∣P04745	Salivary alpha-amylase	1.25	0.00	1.12
98	40	spt∣O75594	Peptidoglycan recognition protein	0.56	0.00	1.08
99	127	emb∣CAA40946.1	Immunoglobulin lambda light chain	1.71	0.01	1.47
100	15	trm∣Q9UJ36	Transmembrane glycoprotein	0.74	0.01	1.25
101	4	gb∣AAH17802.1	SPRR3 protein	0.14	0.00	1.25
102	9	spt∣Q01469	Fatty acid-binding protein	0.29	0.00	1.34
103	4	trm∣Q6FGL5	LCN2 protein	1.36	0.01	1.33
104	10	trm∣Q9UMV3	MBL-associated serine protease 2	0.29	0.00	1.34
105	10	gb∣AAH30653.1	Cadherin 13, preproprotein	2.91	0.00	1.76
106	6	spt∣Q9H8L6	Multimerin 2	0.69	0.02	1.34
107	33	trm∣Q9UD19	Intron-containing kallikrein	0.73	0.02	1.30
108	4	spt∣P07195	L-lactate dehydrogenase B chain	0.75	0.00	1.16
109	9	spt∣P08185	Corticosteroid-binding globulin	3.22	0.00	1.79
110	14	trm∣Q5UGI3	Ubiquitin C splice variant	1.37	0.01	1.24
111	6	pdb∣1O1P_D	D Chain D, Deoxy Hemoglobin	0.55	0.00	1.32
112	7	spt∣P80723	Brain acid soluble protein 1	2.24	0.00	1.46
113	7	spt∣P19320	Vascular cell adhesion protein 1	1.25	0.03	1.23
114	6	spt∣P27797	Calreticulin	1.39	0.00	1.13
115	10	spt∣Q01459	Di-N-acetylchitobiase	1.43	0.01	1.26
116	6	trm∣Q6PN97	Alpha 2 macroglobulin	3.04	0.00	1.84
117	5	gb∣AAV40827.1	superoxide dismutase 3	0.48	0.00	1.15
118	3	spt∣P08473	Neprilysin	2.26	0.00	1.51
119	61	trm∣Q9Y5Y7	LYVE-1	1.63	0.00	1.03
120	2	spt∣P26038	Moesin	1.56	0.00	1.18
121	9	trm∣Q6PIJ0	FCGR3A protein	0.77	0.00	1.06
122	7	spt∣P14209	T cell surface glycoprotein E2	2.02	0.02	1.61
123	6	spt∣P02765	Alpha-2-HS-glycoprotein	1.69	0.00	1.20
124	215	pir∣A23746	Ig kappa chain V-III	1.31	0.00	1.14
125	3	trm∣Q9NT71	Hypothetical protein DKFZp761A051	0.74	0.03	1.31
126	6	trm∣Q9Y4W4	Type XV collagen	0.52	0.00	1.25
127	5	cra∣hCP42501.1	Complement component 1	0.74	0.00	1.20
128	11	spt∣P09564	T-cell antigen CD7	0.67	0.01	1.26
129	6	dbj∣BAA86053.1	Carboxypeptidase E	0.79	0.02	1.20
130	6	spt∣P15151	Poliovirus receptor	1.46	0.01	1.51
131	13	trm∣Q8IUP2	Protocadherin 1, isoform 1	0.49	0.02	1.75
132	3	trm∣Q8NBK0	Hypothetical protein	1.31	0.01	1.19
133	5	spt∣P22891	Vitamin K-dependent protein Z	0.76	0.01	1.21
134	6	trm∣Q9UNF4	Hyaluronic acid receptor	1.50	0.01	1.29
135	11	trm∣Q9HCU0	Tumor endothelial marker 1	0.46	0.00	1.36
136	8	spt∣P35527	Keratin, type I cytoskeletal 9	0.55	0.00	1.29
137	3	spt∣P55285	Cadherin-6	0.41	0.00	1.39
138	3	trm∣Q9BYH7	Scavenger receptor with C-type lectin type I	1.39	0.01	1.23
139	2	trm∣Q13942	Calmodulin	2.64	0.00	1.39
140	8	spt∣P04004	Vitronectin	1.87	0.00	1.42
141	11	trm∣Q86Z23	Hypothetical protein	2.11	0.01	1.49
142	140	trm∣Q9NWE3	Hypothetical protein FLJ10084	1.55	0.00	1.04
143	99	trm∣Q9NWE3	Hypothetical protein FLJ10084	1.53	0.00	1.06
144	11	spt∣P05109	Calgranulin A	0.42	0.00	1.34
145	58	trm∣Q9NWE3	Hypothetical protein FLJ10084	1.50	0.00	1.08
146	3	trm∣Q9BYH7	Scavenger receptor with C-type lectin type I	1.65	0.00	1.24
147	4	gb∣AAA52018.1	Chromogranin A	0.52	0.00	1.26
148	4	emb∣CAI20248.1	PPGB	1.51	0.00	1.24
149	92	pir∣S12443	Ig lambda chain (Ke+O−)−human	1.35	0.03	1.24
150	7	trm∣Q5TEQ5	OTTHUMP00000044363	0.29	0.00	1.58
151	18	trm∣Q6UX86	GPPS559	2.08	0.01	1.60
152	2	trm∣Q8TCZ2	MIC2L1	0.80	0.01	1.16
153	5	spt∣P61970	Nuclear transport factor 2	0.78	0.01	1.18
154	284	pdb∣1T04_C	Anti-ifn-gamma fab in C2 space group	1.45	0.02	1.28
155	8	spt∣P00790	Pepsin A	0.80	0.00	1.15
156	2	cra∣hCP1778903.1	CD7 antigen	0.48	0.00	1.03
157	5	trm∣O00480	Butyrophilin, subfamily 2	0.68	0.00	1.12
158	5	trm∣O43653	Prostate stem cell A	1.70	0.00	1.25
159	4	trm∣Q9BX83	Hemoglobin alpha 1 globin chain	0.67	0.05	1.47
160	9	spt∣P11684	Uteroglobin	3.16	0.00	1.86
161	7	trm∣Q7LDY7	Alpha-KG-E2	1.47	0.00	1.17
162	3	trm∣Q9BYH7	Scavenger receptor	1.92	0.00	1.25
163	4	trm∣Q6AZK5	KRT13 protein	0.56	0.02	1.58
164	5	spt∣P09619	PDGF-R-beta	0.66	0.01	1.29
165	5	pdb∣5TTR_H	Leu 55 Pro Transthyretin	1.46	0.02	1.33
166	3	rf∣NP_877418.1	Mucin 1, transmembrane	0.49	0.02	1.78
167	2	trm∣Q5SWW9	OTTHUMP00000060590	0.39	0.01	1.76
168	2	rf∣NP_003217.2	Trefoil factor 3	1.63	0.00	1.22
169	3	spt∣Q99574	Neuroserpin	0.63	0.01	1.36
170	21	trm∣Q9Y3U9	Hypothetical protein DKFZp566C243	0.75	0.00	1.06
171	23	gb∣AAB27607.1	Prostaglandin D synthase	1.20	0.00	1.10
172	4	gb∣AAO11857.1	Immunoglobulin	1.38	0.00	1.14
173	2	trm∣Q5VTA6	Cubilin	0.79	0.03	1.23
174	3	trm∣Q5SY67	OTTHUMP00000059857	0.69	0.02	1.33
175	54	spt∣P15814	Immunoglobulin lambda-like polypeptide 1	2.50	0.01	1.37
176	2	spt∣P22352	Plasma glutathione peroxidase	0.70	0.00	1.13
177	7	gb∣AAL68978.1	Mutant beta globin	0.32	0.00	1.77
178	6	spt∣P35908	KCytokeratin 2e	0.50	0.03	1.74
179	284	dbj∣BAB18261.1	Anti-HBs antibody light chain	3.19	0.00	1.53
180	3	rf∣XP_370615.2	Hypothetical protein	0.53	0.03	1.72
181	164	gb∣AAB50880.2	Anitubulin IgG1 kappa VL chain	1.64	0.00	1.30
182	170	dbj∣BAC01692.1	Immunoglobulin kappa light chain	2.49	0.01	1.79
183	2	trm∣Q7RTN9	Type II keratin K6h	0.43	0.01	1.52
184	3	spt∣P21926	Motility-related protein	1.70	0.03	1.45
185	2	spt∣Q13873	Bone morphogenetic protein receptor	0.61	0.01	1.08
186	4	gb∣AAA62175.1	Heat shock protein 27	0.53	0.00	1.38
187	15	spt∣P02787	transferrin	1.86	0.00	1.25
188	2	spt∣P07108	Acyl-CoA-binding protein	2.20	0.02	1.47
189	2	spt∣Q9NZH0	G protein-coupled receptor family C	1.27	0.04	1.25
190	2	trm∣Q96E46	Fructose-1,6-bisphosphatase 1	0.44	0.00	1.01
191	44	gb∣AAB53267.1	Immunoglobulin V-region light chain	1.40	0.03	1.30
192	8	gb∣AAR32503.1	Immunoglobulin heavy chain	0.46	0.00	1.19
193	7	rf∣NP_653247.1	Immunoglobulin J chain	0.37	0.01	1.88
194	29	emb∣CAA12585.1	Ig heavy chain variable region	1.50	0.00	1.13
195	4	gb∣AAD16731.1	Immunoglobulin lambda light chain	1.58	0.00	1.11
196	33	trm∣Q6UXB8	HGSC289 (OTTHUMP00000039678)	1.27	0.04	1.22

^a^The numbers of unique peptides and MS/MS spectrum observed by ProteinPilot software were determined only for those peptides with ≥95% confidence. ^b^Accession numbers represent entries in the Human CDS database (human KBMS 5.0, 2005-03-02; a total of 187,748 entries provided by Applied Biosystems). ^c–e^The iTRAQ ratio, *P* value, and EF value in microalbumiuric versus normoalbuminuric urine, respectively.

## Appendices

### A. Differentially Excreted Urinary Proteome in Microalbumiuric versus Normoalbuminuric Urine

For more details, see Table 5.

### B. Mascot Search and ROC Curves for Each Transition of MRM

To verify the MRM validation, we analyzed minimum of 2 transitions for each biomarker candidate protein.

Results from Mascot search and ROC curves for each transition are summarized in Figures 9, 10, 11, 12, 13, 14, 15, 16, 17, 18, and 19.
